# Development of a 63K SNP Array for Cotton and High-Density Mapping of Intraspecific and Interspecific Populations of *Gossypium* spp.

**DOI:** 10.1534/g3.115.018416

**Published:** 2015-04-22

**Authors:** Amanda M. Hulse-Kemp, Jana Lemm, Joerg Plieske, Hamid Ashrafi, Ramesh Buyyarapu, David D. Fang, James Frelichowski, Marc Giband, Steve Hague, Lori L. Hinze, Kelli J. Kochan, Penny K. Riggs, Jodi A. Scheffler, Joshua A. Udall, Mauricio Ulloa, Shirley S. Wang, Qian-Hao Zhu, Sumit K. Bag, Archana Bhardwaj, John J. Burke, Robert L. Byers, Michel Claverie, Michael A. Gore, David B. Harker, Md S. Islam, Johnie N. Jenkins, Don C. Jones, Jean-Marc Lacape, Danny J. Llewellyn, Richard G. Percy, Alan E. Pepper, Jesse A. Poland, Krishan Mohan Rai, Samir V. Sawant, Sunil Kumar Singh, Andrew Spriggs, Jen M. Taylor, Fei Wang, Scott M. Yourstone, Xiuting Zheng, Cindy T. Lawley, Martin W. Ganal, Allen Van Deynze, Iain W. Wilson, David M. Stelly

**Affiliations:** * Department of Soil & Crop Sciences, Texas A&M University, College Station, Texas 77843; † Interdisciplinary Degree Program in Genetics, Texas A&M University, College Station, Texas 77843; ‡ TraitGenetics GmbH, 06466 Gatersleben, Germany; § Department of Plant Sciences and Seed Biotechnology Center, University of California-Davis, Davis, California 95616; ** Dow AgroSciences, Trait Genetics and Technologies, Indianapolis, Indiana 46268; †† USDA-ARS-SRRC, Cotton Fiber Bioscience Research Unit, New Orleans, Louisiana 70124; ‡‡ USDA-ARS-SPARC, Crop Germplasm Research Unit, College Station, Texas 77845; §§ CIRAD, UMR AGAP, Montpellier, F34398, France; *** EMBRAPA, Algodão, Nucleo Cerrado, 75.375-000 Santo Antônio de Goias, GO, Brazil; ††† Department of Animal Science, Texas A&M University, College Station, Texas 77843; ‡‡‡ USDA-ARS, Jamie Whitten Delta States Research Center, Stoneville, Mississippi 38776; §§§ Brigham Young University, Plant and Wildlife Science Department, Provo, Utah 84602; **** USDA-ARS, PA, Plant Stress and Germplasm Development Research Unit, Lubbock, Texas 79415; †††† CSIRO Agriculture Flagship, Black Mountain Laboratories, ACT 2601, Australia; ‡‡‡‡ CSIR-National Botanical Research Institute, Plant Molecular Biology Division, Lucknow-226001, UP, India; ‡‡‡‡‡ Department of Biology, Texas A&M University, College Station, Texas 77843; §§§§ Plant Breeding and Genetics Section, School of Integrative Plant Science, Cornell University, Ithaca, New York 14853; ***** USDA-ARS, Genetics and Precision Agriculture Research, Mississippi State, Mississippi 39762; ††††† Cotton Incorporated, Agricultural Research, Cary, North Carolina 27513; §§§§§ Wheat Genetics Resource Center, Department of Plant Pathology and Department of Agronomy, Kansas State University, Manhattan, Kansas 66506; ****** Illumina Inc., San Francisco, California 94158

**Keywords:** linkage analysis, recombination, interspecific SNPs, intraspecific SNPs, breeding

## Abstract

High-throughput genotyping arrays provide a standardized resource for plant breeding communities that are useful for a breadth of applications including high-density genetic mapping, genome-wide association studies (GWAS), genomic selection (GS), complex trait dissection, and studying patterns of genomic diversity among cultivars and wild accessions. We have developed the CottonSNP63K, an Illumina Infinium array containing assays for 45,104 putative intraspecific single nucleotide polymorphism (SNP) markers for use within the cultivated cotton species *Gossypium hirsutum* L. and 17,954 putative interspecific SNP markers for use with crosses of other cotton species with *G. hirsutum*. The SNPs on the array were developed from 13 different discovery sets that represent a diverse range of *G. hirsutum* germplasm and five other species: *G. barbadense* L., *G. tomentosum* Nuttal × Seemann, *G. mustelinum* Miers × Watt, *G. armourianum* Kearny, and *G. longicalyx* J.B. Hutchinson and Lee. The array was validated with 1,156 samples to generate cluster positions to facilitate automated analysis of 38,822 polymorphic markers. Two high-density genetic maps containing a total of 22,829 SNPs were generated for two F_2_ mapping populations, one intraspecific and one interspecific, and 3,533 SNP markers were co-occurring in both maps. The produced intraspecific genetic map is the first saturated map that associates into 26 linkage groups corresponding to the number of cotton chromosomes for a cross between two *G. hirsutum* lines. The linkage maps were shown to have high levels of collinearity to the JGI *G. raimondii* Ulbrich reference genome sequence. The CottonSNP63K array, cluster file and associated marker sequences constitute a major new resource for the global cotton research community.

Cotton (*Gossypium* spp.) is the world’s most important renewable natural textile fiber crop and also a significant source of oilseed. Cotton is grown in more than 75 countries, with more than 118 million bales of cotton fiber produced in 2013 (National Cotton Council, www.cotton.org). In the United States, the 2013 crop of 12.9 million bales was valued at $5.2 billion and had an estimated overall direct economic impact of $27.6 billion (US Department of Agriculture; National Agriculture Statistics Service, www.nass.usda.gov). Approximately 75% of cotton fiber is used for apparel products, 18% is used for home furnishings, and 7% is used for industrial products (National Cotton Council, www.cotton.org). In the past, cottonseed was often regarded as waste, but recently it has become marketable as a protein source for livestock, including dairy cattle and poultry, as well as for production of cottonseed oil, which is used in the food product industry . Although 52 different *Gossypium* species have been identified, including 7 tetraploids designated AD_#_ and 45 diploids classified into 8 genome designations (A–G and K) (Wendel *et al.* 2009; Grover *et al.* 2014), contemporary cotton production relies primarily on allotetraploid *Gossypium hirsutum* L. (2*n* = 4*x* = 52), which accounts for more than 95% of the world crop. The remaining production relies largely on *G. barbadense* L., another allotetraploid, and to a lesser extent on two A-genome diploids (2*n* = 2*x* = 26), *G. arboreum* L. and *G. herbaceum* L., primarily in Asia. Although diploid species of interest such as *G. longicalyx*, an F-genome diploid, and *G. armourianum*, a D-genome diploid, cannot be readily crossed with the primary cultivated species *G. hirsutum*, allotetraploid species such as *G. barbadense*, *G. tomentosum*, and *G. mustelinum* can be readily intercrossed.

Allotetraploid cotton was formed approximately 1–2 million years ago (mya) (Wendel *et al.* 2009) in a polyploidization event between two diploids: one with an A-like genome and the other with a D-like genome. The ancestral genomes resemble the genomes of extant diploids *G. arboreum* (A_2_) and *G. raimondii* (D_5_). The *G. hirsutum* genome is designated [AD]_1_ and has a C-value of approximately 2.4 Gbp; the 26 chromosomes are numbered according to genome ancestry, where chromosomes 1–13 are of A-genome origin and 14–26 are of D-genome origin (Brown 1980; Wendel *et al.* 2009). Because the ancestral genomes of cotton diverged only 5–10 mya, and because the polyploidization event was 1–2 mya, initial efforts to develop single nucleotide polymorphism (SNP) markers were hindered by the co-identification of interlocus SNP variants between the two subgenomes in the tetraploid (homeo-SNPs). Recent developments from the cotton community including the publication of a high-quality genome reference sequence for *G. raimondii* (Paterson *et al.* 2012) and draft sequences for *G. arboreum* (Li *et al.* 2014) and *G. raimondii* (Wang *et al.* 2012) have aided development of large SNP data sets in gene-based and genome-based identification efforts (Van Deynze *et al.* 2009; Byers *et al.* 2012; Lacape *et al.* 2012; Rai *et al.* 2013; Gore *et al.* 2014; Islam *et al.* 2014; Zhu *et al.* 2014; Hulse-Kemp *et al.* 2014; Hulse-Kemp *et al.* 2015). Increasingly larger genetic maps with greater resolution and saturation have been generated as numbers of primarily SSR markers increased, culminating in the recent publication of a consensus genetic map that comprises more than 8,200 loci based primarily on six interspecific (*G. hirsutum* × *G. barbadense*) populations (Blenda *et al.* 2012). Few of the published genetic maps contain SNP markers, and those that have included SNPs are limited to inclusion of a few hundred SNPs with SSRs (Byers *et al.* 2012; Yu *et al.* 2012; Gore *et al.* 2014) to, at most, the inclusion of a few thousand SNPs (Islam *et al.* 2014; Zhu *et al.* 2014). SNPs have been difficult to develop *in silico* due to low polymorphism and low divergence among polyploid genomes (Van Deynze *et al.* 2009). The increasing efficiency of next-generation sequencing and improved *in silico* methods has allowed SNP development at the whole genome level, even for the 2.4-Gbp allotetraploid cotton genome (Hulse-Kemp *et al.* 2015; Zhu *et al.* 2014).

In cultivated cotton, as well as in other crop species, there is considerable interest in being able to genotype a large number of SNP markers in a high-throughput manner. With advancing array technology, large-scale genotyping can be performed in a massively parallel fashion to assay thousands of loci simultaneously in a short time. Genotype data obtained from the high-throughput assay can then be utilized to evaluate genetic diversity, construct genetic linkage maps, dissect the genetic architecture of important traits (Truco *et al.* 2013), and in many more novel applications (Ganal *et al.* 2012). SNP arrays for many crop species have recently been developed and have been utilized to advance breeding and discoveries in those crop systems (Ganal *et al.* 2011; Hamilton *et al.* 2011; Truco *et al.* 2013; Chen *et al.* 2013; Song *et al.* 2013; Tinker *et al.* 2014; Bianco *et al.* 2014; Wang *et al.* 2014; Dalton-Morgan *et al.* 2014).

The objectives of this study were as follows: to utilize recently identified SNPs to develop a standardized large-scale SNP genotyping array for cotton; to evaluate the performance and reproducibility of the array on a large set of samples; to develop a cluster file that can be used to help automate genotyping for allotetraploid cotton; and to produce high-density linkage maps based on two biparental F_2_ populations in an intraspecific cross (*G. hirsutum* × *G. hirsutum*) and an interspecific cross (*G. barbadense* × *G. hirsutum*). Completion of these objectives will enable reliable standardized high-throughput SNP genotyping on large cotton populations for many diverse applications, including high-density genetic mapping, genome-wide associated studies, genomic selection, complete trait dissection, and studying patterns of genomic diversity among cotton cultivars and wild accessions.

## Materials and Methods

### Plant materials

Seeds for each line were planted in peat pellets (Jiffy, Canada) at Texas A&M University, CIRAD/EMBRAPA, USDA-ARS-SPARC, USDA-ARS-SRRC, or CSIRO greenhouses. Plants were allowed to grow until first true leaves were available. Young true leaves were sampled and extracted using the Macherey-Nagel Plant Nucleo-spin kit (Pennsylvania) according to the manufacturer’s instructions. All DNA samples were quantified using PicoGreen and then diluted to 50 ng/µl. The samples included reference lines from the various SNP development efforts, duplicated DNA samples, individual plants and plant pools from the same seed source, individual plant samples from different seed sources, parent/F_1_ combinations, segregating samples from wild *Gossypium* species, inbred cultivar lines, wild *G. hirsutum* lines, and two mapping populations, one intraspecific and one interspecific (Table 1). This material represented samples from most of the cross-compatible range of *G. hirsutum*. Mapping samples included 93 F_2_ lines from a *G. hirsutum* cv. Phytogen 72 × *G. hirsutum* cv. Stoneville 474 population designated as “PS” for intraspecific mapping and 118 F_2_ lines from a *G. barbadense* doubled haploid line 3-79 × *G. hirsutum* cv. Texas Marker-1 population designated as “T3” for interspecific mapping. Samples for the mapping populations were chosen randomly from germinated seed within each population.

**Table 1:** Samples included for array validation and cluster file development

Sample Type	No. of Samples
Inbred, *G. hirsutum* (cultivated)	516
Inbred, *G. hirsutum* (wild)	59
Intraspecific F_1_	53
Intraspecific F_2_	157
Intraspecific backcross	31
Intraspecific RIL	34
Inbred, *G. barbadense*	18
Interspecific F_2_	69 (49*^a^*)
Interspecific RIL	14
Interspecific aneuploid	21*^b^*
Wild tetraploid species	4
Synthetic tetraploid	3
Diploid species	8*^b^*
Interspecific backcross	146
Interspecific F_1_	20
Haploid	3*^b^*
Total	1156

a A total of 49 interspecific F_2_ samples were not included in cluster file development but were genotyped using the resulting cluster file for inclusion in linkage mapping.

b These samples were used in the cluster file development, but the cluster file is not suitable for scoring such samples because it is only optimized for tetraploid samples.

### Array design

SNP data sets were obtained for nine intraspecific (Table 2) and four interspecific SNP development efforts (Table 3). Classification of SNPs into intraspecific or interspecific content on the array was based on where the SNP was identified in our data sets (Table 2 and Table 3). The datasets obtained included SNPs from the following: restriction enzyme double-digest procedure in *G. hirsutum* between a cultivated and wild accession (Byers *et al.* 2012); long-read 454 sequencing of restriction-based genic enrichment libraries of six *G. hirsutum* lines (Rai *et al.* 2013); genotyping-by-sequencing (GBS) mapping of a cross between two *G. hirsutum* lines (Gore *et al.* 2014); transcriptome sequencing of five *G. hirsutum* lines (Ashrafi *et al.* 2015); GBS mapping of a multi-parent population (Islam *et al.* 2014); transcriptome sequencing and restriction-based analysis of 18 *G. hirsutum* lines (Zhu *et al.* 2014); re-sequencing of 12 *G. hirsutum* lines (Hulse-Kemp *et al.* 2015) (H. Ashrafi *et al*., personal communication); unclassified markers mapped to known chromosomes (DOW AgroSciences, unpublished data); single-copy sequence-derived SNPs between *G. hirsutum* and *G. barbadense* (Van Deynze *et al.* 2009); differential expression analysis using RNA-sequencing of *G. hirsutum* vs. *G. barbadense* (Lacape *et al.* 2012); transcriptome sequencing of *G. barbadense*, *G. tomentosum*, *G. mustelinum*, *G. armourianum*, and *G. longicalyx* (Hulse-Kemp *et al.* 2014); and re-sequencing of *G. barbadense* (Hulse-Kemp *et al.* 2015).

**Table 2:** Datasets utilized in intraspecific content design on the CottonSNP63K array

Data Set Name	Authors/Reference	Lines
Brigham Young University	Byers *et al.* (2012)	Acala Maxxa, TX2094
CSIR-NBRI	Rai *et al.* (2013)	JKC703, JKC725, JKC737, JKC770, MCU-5, LRA5166
USDA Set 1	Gore *et al.* (2014)	TM-1, NM24016
UC-Davis/TAMU GH RNA-seq	Ashrafi *et al.* (2015)	TM-1, FM832, Sealand 542, PD-1, Acala Maxxa
USDA Set 2	Islam *et al.* (2014)	Acala Ultima, Pyramid, Coker 315, STV825, FM966, M-240 RNR, HS26, DP-90, SG747, PSC355, STV474
CSIRO	Zhu *et al.* (2014)	MCU-5, Delta Opal, Sicot 70, Siokra 1-4, DP-16, Tamcot SP37, Namcala, Riverina Poplar, LuMein 14, Sicala 3-2, Sicala 40, Sicala V-2, Sicot 81, Sicot 71, Sicot 189, Sicot F-1, Deltapine 90, Coker 315
TAMU/UC-Davis Intra Genomic Set 1	Hulse-Kemp *et al.* (2015)	M-240 RNR, TM-1, HS26, SG747, STV474, FM832, Sealand542, PD-1, Coker 312, Tamcot Sphinx, TX231, Acala Maxxa
UC-Davis/TAMU Intra Genomic Set 2	Ashrafi *et al.* (unpublished)	M-240 RNR, TM-1, HS26, SG747, STV474, FM832, Sealand542, PD-1, Coker 312, Tamcot Sphinx, TX231, Acala Maxxa
DOW AgroSciences	DAS (unpublished)	Unreleased

A total of 50K putative single nucleotide markers were used to produce the 45,104 intraspecific assays on the array after production. DAS, DOW AgroSciences.

**Table 3:** Datasets utilized in inter-specific content design the CottonSNP63K array

Data Set Name	Authors/Reference	Lines
UC-Davis Inter	Van Deynze *et al.* (2009)	*G. barbadense* (3-79), *G. hirsutum* (TM-1)
CIRAD	Lacape *et al.* (2012)	*G. barbadense* (VH8-4602), *G. hirsutum* (Guazuncho II)
TAMU/UC-Davis Inter RNA-seq	Hulse-Kemp *et al.* (2014)	*G. barbadense* (3-79), *G. tomentosum*, *G. mustelinum*, *G. armourianum*, *G. longicalyx*
TAMU/UC-Davis Inter Genomic	Hulse-Kemp *et al.* (2015)	*G. barbadense* (3-79)

A total of 20K putative single nucleotide markers were used to produce the 17,954 inter-specific assays on the array after production.

The Illumina Infinium technology utilizes a bead-based approach in which 50 bp oligonucleotide probes are used to hybridize to SNP-adjacent sequences of a sample and then a single base pair extension is performed to assay the SNP base with fluorescently labeled nucleotides. The technology utilizes a two-fluorophore system, necessitating the use of two beadtypes to discriminate only those SNPs that target alleles sharing the same fluorophore (transversions) and only one beadtype to capture all other SNP types (transitions). Based on the Infinium technology, putative markers were filtered to maximize value from this technology, *i.e.*, to select for unique one-beadtype assays to populate the array. Putative SNPs were retained based on requirements of design score >0.8 (Illumina, Inc), unique identity >99% over 100 bp of flanking sequence, >99% unique identity for designed probe sequence, with final SNPs retained based on precedent publication order in the hierarchy listed above. Subsequent filtering steps included prioritization for the following: one beadtype (Infinium II) assays; validated markers; experimental screening success rates; and representation of genic and nongenic SNPs to optimally cover the cotton genome to obtain a final set of 70,000 putative SNP markers for inclusion on the array (Supporting Information, Table S1). All genic-based data sets were filtered during dataset development to eliminate SNPs near predicted intron boundaries (Hulse-Kemp *et al.* 2014; Ashrafi *et al.* 2015; Zhu *et al.* 2014) so SNPs and flanking sequences could be aligned directly with genomic-based markers to filter putative SNPs as indicated above.

**Figure 1:**
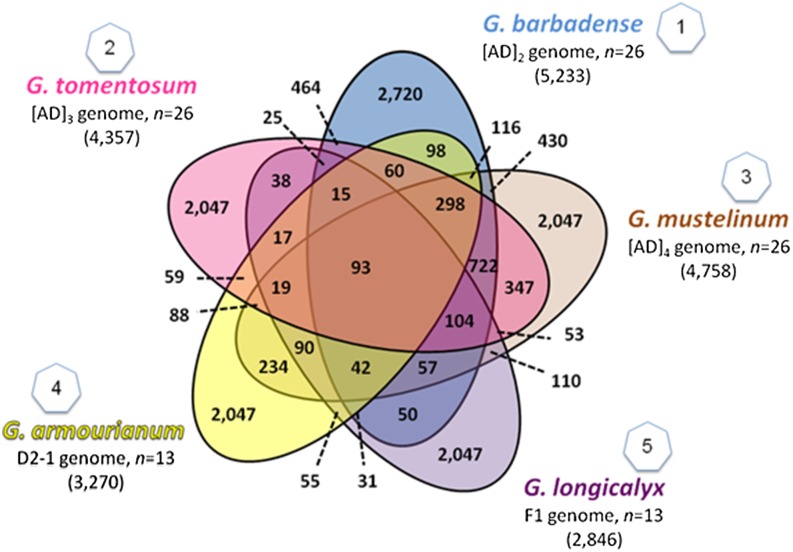
SNP markers shared across five species included on the CottonSNP63K array from TAMU/UC-Davis Inter RNA-seq discovery set (Hulse-Kemp *et al.* 2014).

Due to limited prior validation, 500 markers were randomly selected for inclusion from the UC-Davis/TAMU Genomic Set 2 and 386 markers were selected from the CSIR-NBRI data set, including 252 that were chosen randomly and 134 markers that were selected for inclusion based on overlap as identified with BLAST to have 100% identity with markers from other data sets over the entire length of SNP and flanking sequences. For USDA Set 2, markers were primarily included for contigs that had low missing data and were detected across multiple lines with minor allele frequency greater than 0.1. The markers chosen from the CIRAD set primarily represent markers from the most highly differentially expressed transcripts identified between *G. hirsutum* and *G. barbadense*. From the TAMU/UC-Davis Inter RNA-Seq data set, as many SNPs as possible that are common between the five species represented and *G. hirsutum* were selected for inclusion, as well as sets of private SNPs unique to each of the five species (Figure 1).

### Genotyping with the array

Standardized DNA at 50 ng/µl for each of the cotton lines described above was processed according to Illumina protocols and hybridized to the CottonSNP63K array at Texas A&M University or CSIRO. Single-base extension was performed and the chips were scanned using the Illumina iScan. Image files were saved for cluster file analysis. All image files were uploaded into a single GenomeStudio project containing 1156 individual samples. Of the 70,000 SNPs targeted for manufacture on the array, 6942 markers failed to meet standards for bead representation and decoding metrics during the array construction process at Illumina and were removed from the manifest. Data for the remaining markers were clustered using the GenomeStudio Genotyping Module (V 1.9.4, Illumina, Inc.). All markers were viewed and manually curated, taking into account the sample type and known segregation ratios, for construction of the best cluster file for genotyping tetraploid cotton (available at http://www.cottongen.org/node/add/cotton-cluster-file-request).

### Reproducibility and call rate in a diverse set of cotton samples

Three technical replicates (running the same DNA on different genotyping runs) were processed for an individual DNA sample of *G. hirsutum* cultivar TM-1, *G. barbadense* inbred line 3-79 and for the F_1_ individual from a *G. hirsutum* (TM-1) by *G. barbadense* (3-79) cross. Different individual plants from the same line (cultivar or accession) were analyzed for 11 lines where the individual plants were obtained from the same seed source and for 11 lines where the individual plants were obtained from different seed sources. Genotypes from these lines were compared to determine similarity across technical replicates, individuals from different seed sources, and individuals from the same seed source. Multiple individuals (12) were pooled into the same DNA sample and then compared to the genotype from a single individual to determine variability within a seed source as well as percent residual heterozygosity. Polymorphic SNPs were classified based on Illumina GenTrain score (proximity of clusters) and call frequencies across samples. Minor allele frequencies of polymorphic markers were determined using only inbred line samples.

### Genetic linkage analysis: intraspecific and interspecific

Genotyping data were transformed into mapping data format (“ABH”) for the 93 intraspecific PS F_2_ samples and the 118 interspecific T3 F_2_ samples. Only markers that had opposite homozygous allele calls between parental samples and behaved co-dominantly were retained. Subsequently, the data files were initially mapped with JoinMap 4.0 (Van Ooijen 2006) with respect to verification of segregation patterns, the formation of linkage groups, and the preliminary map position of the markers on the chromosomes using the default grouping settings and the maximum likelihood mapping algorithm.

The map data resulting from the initial mapping analysis were manually curated to identify and remove problematic markers that caused elevated numbers of double crossovers and expansions in the length of the linkage group. After removing the problematic markers from the ABH mapping data file in Microsoft Excel, the revised ABH mapping data file was used to construct the final linkage groups in MapManager QTX (Manly, Cudmore, and Meer 2001) with the following settings: linkage evaluation F_2_, search linkage criterion *P* = 0.05, map function Kosambi, and line cross cross-type. Markers were eliminated from the final mapping if they caused unexpected expansion of the linkage group because of too many crossovers because this is likely the result of low scoring quality of the individual marker analysis, *i.e.*, markers with low GenTrain score and/or call frequency. The final maps were drawn with MapChart version 2.2 (Voorrips 2002) with one marker per centimorgan (cM) to allow easier visualization; positions for all markers are listed in Table S1. Linkage disequilibrium and visualization of crossovers for each determined linkage group were plotted using the CheckMatrix software (www.atgc.org/XLinkage/) to analyze the mapping population genotypes. The correlation between the interspecific and intraspecific map orders was visualized using MapChart. Discordant linkage groups in the interspecific map identified using CheckMatrix and MapChart were remapped in MapManager using a framework map from the corresponding intraspecific linkage group. The number of recombination events per each F_2_ individual was calculated for both the intraspecific and the reordered interspecific linkage maps. Average numbers of recombination bins across both linkage maps were also calculated. Recombination bins were considered as the interval between one recombination breakpoint and the next breakpoint within the mapping populations.

### Synteny analyses

All SNP marker sequences were aligned to the high-quality JGI *G. raimondii* (D_5_) reference genome (Paterson *et al.* 2012) using Burrows Wheeler Alignment (BWA) software in GALAXY (Goecks, Nekrutenko, and Taylor 2010) with default parameters. Linkage map positions were plotted against D_5_ alignment position for both the interspecific and intraspecific mapped markers. The corresponding allotetraploid chromosomes of the linkage groups were identified using mapped markers (Dow AgroSciences, unpublished data) (Yu *et al.* 2014; Blenda *et al.* 2012) and D_5_ alignment information. Linkage groups were oriented using the cotton consensus map that incorporated SSR marker chromosome assignment information available (Blenda *et al.* 2012). All markers were also aligned to the BGI *G. arboreum* (A_2_) draft sequence (Li *et al.* 2014) and plotted.

## Results

### Genotyping array content

An Illumina Infinium genotyping array was developed targeting 70,000 putative SNP markers (File S1). The 70,000 putative markers represent 50,000 SNPs that have been identified for use in intraspecific crosses between *G. hirsutum* lines and 20,000 SNPs for use in interspecific crosses between *G. hirsutum* and other *Gossypium* species, such as *G. barbadense*, *G. tomentosum*, *G. mustelinum*, *G. armourianum*, and *G. longicalyx*. The intraspecific set was developed from an initial set of 1,658,397 putative SNP markers; however, the largest data set (UC-Davis/TAMU Intra Genomic Set 2) had not been experimentally validated previously and another large data set (CSIR-NBRI) had limited validation, so only small random samples of SNPs for these sets were included. Therefore, selection of the intraspecific markers for the array was based primarily on 91,953 markers. All markers were submitted through the Illumina Design Tool to determine assay design scores for each marker. The data set was filtered to retain only Infinium II, or one-bead type assays that assay one SNP per bead, and those with design scores greater than 0.8. This resulted in 69,306 SNPs or 75.4% retention. Results of probe design for each of the data sets are shown in Table S2. Duplicated SNPs were eliminated, as noted in *Materials and Methods*. All available SNP markers (18,348) within genes (genic) and a randomly selected set of genomic sequence-derived markers (31,396) were included, for a total of 50,000 intraspecific markers. The resulting intraspecific set comprises 36.7% genic SNPs, 62.8% nongenic SNPs, and 0.5% unclassified SNPs.

The same selection and filtering method was used for the interspecific data set. The SNP selection process started with 314,894 potential markers. After the initial step of filtering based on assay design score and one-bead type markers, there were 234,370 markers. As many previously mapped markers as possible were included. When feasible, a single marker was selected per reference sequence or gene sequence. Additional markers were randomly selected from the filtered data set to complete the final set of 20,000 interspecific assays. A total of 14,689 markers in genes were included and 5311 genomic-derived markers were included. The interspecific final set comprises 73.5% genic SNPs and 26.5% nongenic markers. Within the interspecific set, the TAMU/UC-Davis Inter RNA-seq markers were selected to contain the maximum number of markers with polymorphism across multiple species (relative to *G. hirsutum*) as possible and approximately 2000 markers unique to each of the wild species. This allowed for a maximum number of markers that can be used for introgression breeding efforts while occupying as few beads as possible for cost purposes (Figure 1). Approximately 18% of the interspecific set is composed of markers chosen to support work for multiple species of germplasm introgression into *G. hirsutum*.

Together, the selected intraspecific and interspecific sets resulted in 70,000 SNP markers submitted to manufacturing for inclusion on the array. All of these SNPs have been deposited in the CottonGen database (Yu *et al.* 2014; www.cottongen.org). Based on the characteristics of the utilized Illumina array format, a total of 90,000 beads can be assayed per sample; therefore, it is possible to add up to 20,000 assays, which can be included as private add-on content to allow for adaptability of the array.

### Automated genotype calling through cluster definition

**Figure 2:**
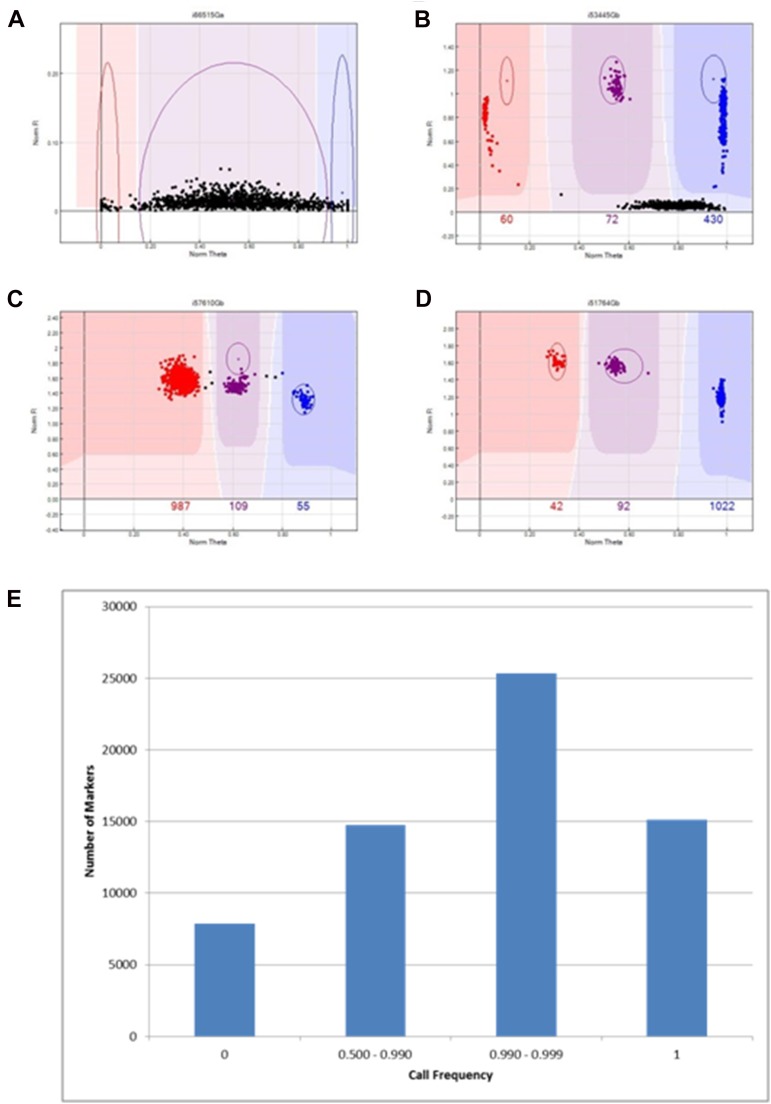
Types of call frequency of SNP markers. NormTheta or relative amount of each of the two fluorophore signals is plotted on the X-axis, whereas NormR or signal intensity is plotted on the Y-axis. (A) Failed marker with call frequency = 0. (B) Call frequency 0.500–0.990 with major sample deviations. (C) Call frequency 0.990–0.999 with few uncalled samples. (D) Call frequency = 1 with all called samples. (E) Distribution of call frequencies for all SNP markers on the array.

From the 70,000 markers sent into production, 63,058 markers passed Illumina manufacturing and quality control and were analyzed on 1156 samples for amenability to automated genotyping. Detailed analyses of the diversity and population structure represented by the germplasm used in this project are underway and will be reported separately. The proportion of samples amenable to genotyping, or “call frequency,” across all markers on the chip could be grouped into four distinct types (Figure 2, A–D) based on all 1156 samples that were included for cluster file development; the distribution of markers within the four types were examined (Figure 2E). Importantly, the polymorphic markers have an average call frequency of 0.99. The first type of classification represents failed markers that did not amplify in the majority of samples (Figure 2A). The second type consists of markers that had many samples with uncalled genotypes and therefore had a call frequency between 0.50 and 0.99 (Figure 2B), which may be caused by the presence of an additional null allele. Type 3 includes markers in which only a few samples remain uncalled (Figure 2C). The last type is those markers in which all samples are called and are highly reproducible (Figure 2D). Call frequency distribution of all synthesized SNP markers on the chip are shown in Figure 2E.

**Figure 3:**
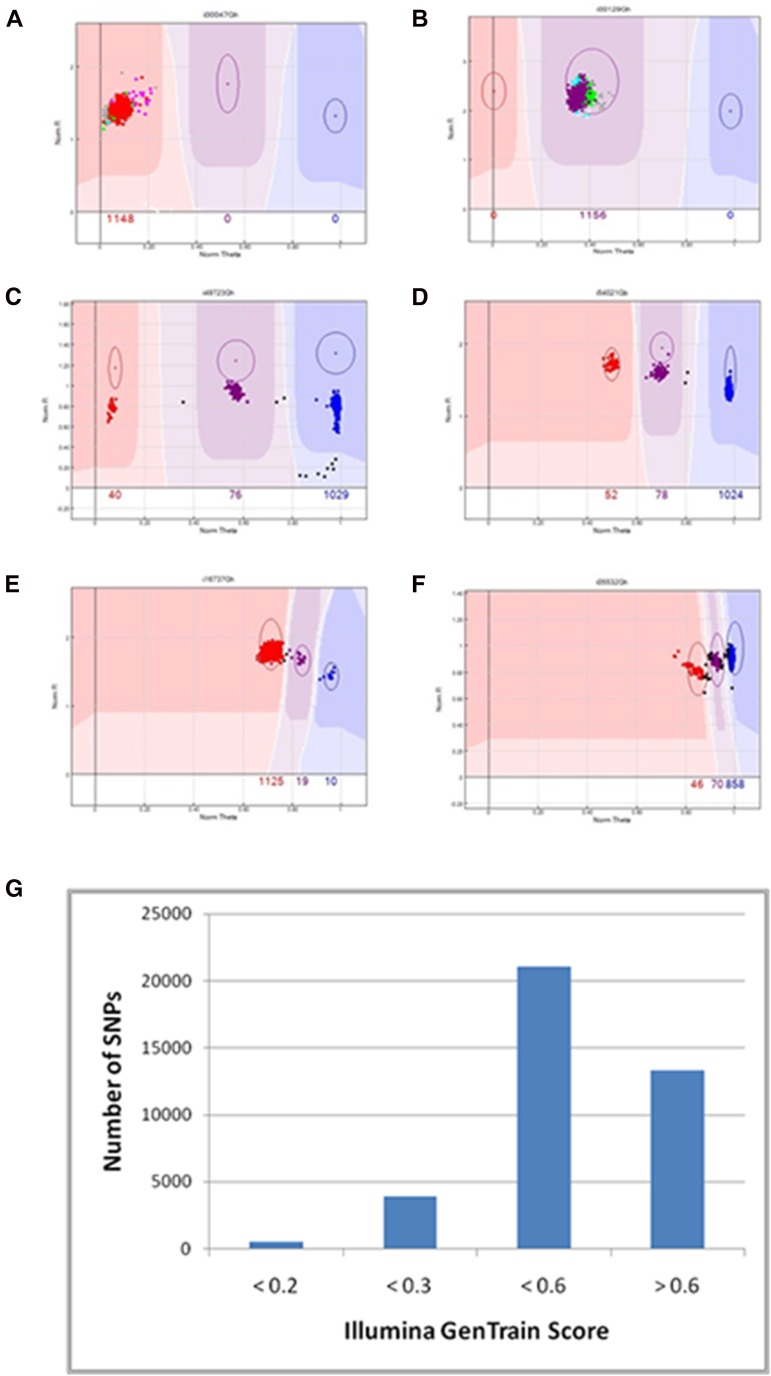
Classification of scorable SNP markers according to Illumina GenTrain score. NormTheta or relative amount of each of the two fluorophore signals is plotted on the X-axis, whereas NormR or signal intensity is plotted on the Y-axis. (A) Monomorphic marker. (B) Intergenomic or homeo-SNP marker. (C–F) Classification of polymorphic markers based on Illumina GenTrain score. (C) Genome-specific marker representing a single polymorphic locus with GenTrain score >0.60. (D) Marker with GenTrain score 0.30–0.59 on half the plot representing two genomes, one monomorphic and one polymorphic locus. (E) Marker with GenTrain score 0.21–0.29 representing multiple monomorphic loci and one polymorphic locus. (F) Marker with GenTrain score less than 0.20 representing many monomorphic loci and one polymorphic locus. (G) Distribution of cluster types in polymorphic markers based on GenTrain score.

Successful markers primarily produced six distinct clustering patterns (Figure 3, A–F). In the first pattern type, essentially all samples fall in a single cluster. This type represents probe sequences that detect a monomorphic locus or loci (Figure 3A). The second pattern type represents markers that are detecting two monomorphic loci, which are each homozygous for a different allele (Figure 3B). Markers in this category appear to be heterozygous in all lines and correspond to intergenomic SNPs or “homeo-SNPs,” which are differences between the two subgenomes of cotton and are often identified as false positives in SNP discovery. Polymorphic markers are attributed to the remaining four patterns; they can be classified according to their GenTrain score. Pattern type 3 are markers that showed three clearly definable clusters and behaved as a traditional co-dominant, diploid-like marker with three possible genotypes (AA, AB, BB) and homozygous genotype clusters located near 0 and 1 (Figure 3C). These types of markers did not require any significant manual adjustment of marker positions. Similarly, the fourth pattern type (Figure 3D) showed three clearly identifiable clusters, but the clusters were shifted toward one side of the plot with one homozygous cluster at 0.5 on the X-axis. Pattern 4 markers represent markers that detect two loci, most likely from the homeologous chromosomes: one polymorphic and one monomorphic resulting in three possible genotypes (AAAA, AAAB, AABB). Pattern 5 markers represent a pattern in which the three clusters are quite close together and likely represent three or more loci with one polymorphic locus and multiple monomorphic loci in the background (Figure 3E). Finally, pattern 6 markers represent markers that show extremely close clusters due to assaying a large number of loci (Figure 3F). Marker patterns 4 and 5 typically required manual adjustment of locus positions, whereas pattern 6 markers were frequently set as failed.

**Figure 4:**
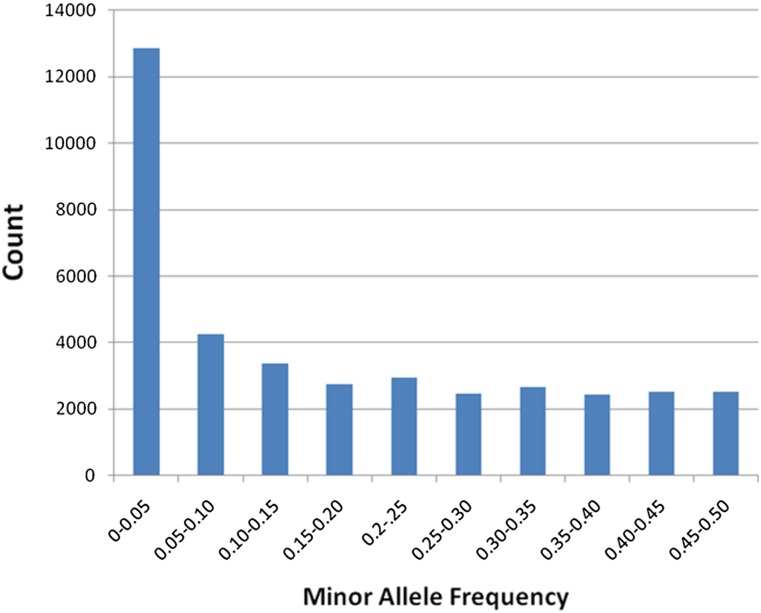
Distribution of minor allele frequencies of all polymorphic SNPs on the CottonSNP63K array. Minor allele frequencies were determined using only inbred line samples; mapping samples and other noninbred line samples used for cluser file development were excluded from this analysis.

After evaluating and manually curating all markers, a total of 55,201 of the markers produced successful assays. The successful assays were further characterized into 38,822 polymorphic markers, 10,314 monomorphic markers, and 6065 intergenomic markers. Thus, the overall success rate of the chip is represented by a minimum of 38,822 polymorphic markers (in the samples assayed) out of the 63,058 markers that were synthesized on the chip (61.6%) that will be useful for genotyping cotton samples. Analysis of minor allele frequency (MAF) across polymorphic markers using only inbred samples showed that 66.8% of the polymorphic markers have MAF above 0.05, 55.8% have MAF above 0.10, and 40.0% have MAF above 0.2. The average MAF among polymorphic markers on the CottonSNP63K array was found to be 0.17. Distribution of minor allele frequencies of polymorphic markers in inbred samples is shown in Figure 4.

Table 4 shows the distribution of the final markers and their success rates across the designed data sets. Although most data sets had a success rate of 50% or more, some had a much lower success rate. Discovery sets that did not have previous validation or had limited validation with PCR-based assays frequently resulted in lower success rates, *e.g.*, CSIR-NBRI and USDA Set 1. In the CSIR-NBRI data set, low success is likely due to homeo-SNPs because it showed the highest percentage of markers classified as 33.82% intergenomic markers. The other data set that had a low success rate of 8.65% was the USDA Set 1 containing markers previously mapped by GBS (Gore *et al.* 2014). Although the CSIR-NBRI data set showed elevated levels of intergenomic markers, the USDA Set 1 was primarily found to have monomorphic markers (77.88%). Because these markers had been previously mapped, and because their map positions correlate with alignment positions on JGI D_5_ genome (Gore *et al.* 2014), these are likely true markers that are of very low MAF or specific to the individual bi-parental population used in their identification (Gore *et al.* 2012). Although lines for the parents of the population were included in the study, this population has been shown to contain nonparental derived alleles (Gore *et al.* 2012). Thus, without lines that represent the nonparental derived alleles, polymorphism would not be able to be identified and this would lead to the monomorphic classification of these markers.

**Table 4:** Distribution of classified SNPs across the discovery sets and success rates of these SNPs on the CottonSNP63K array

Data Set	SNPs on Array	Failed		Successful Assays (No.)			Success Rate (%)
		No.	%	Monomorphic	Intergenomic	Polymorphic	
Brigham Young University	185	23	12.43	16	5	141	76.22
CSIR-NBRI	343	41	11.95	84	116	102	29.74
USDA-Set1	104	10	9.62	81	4	9	8.65
UC-Davis/TAMU GH RNA-Seq	938	153	16.31	81	66	638	68.02
USDA Set 2	2223	474	21.32	193	372	1184	53.26
CSIRO	17,230	2048	11.89	4325	772	10,085	58.53
TAMU/UC-Davis Intra Genomic Set 1	23,418	3509	14.98	4639	3565	11,705	49.98
UC-Davis/TAMU Intra Genomic Set 2	445	48	10.79	5	41	351	78.88
DOW AgroSciences	218	13	5.96	1	0	204	93.58
UC-Davis Inter	143	10	6.99	0	1	132	92.31
CIRAD	145	20	13.79	14	27	84	57.93
TAMU/UC-Davis Inter RNA-Seq	13,055	913	6.99	374	307	11,461	87.79
TAMU/UC-Davis Inter Genomic	4611	595	12.90	501	789	2726	59.12
Total	63,058	7857	12.46	10,314	6065	38,822	61.57

### Reproducibility and call rate in different cotton samples

Replicates of samples were analyzed to determine reproducibility across genotyping runs as well as reproducibility across seed sources. To do this, four different types of replicates were used: technical replicates running the same DNA on different genotyping runs; individual plants from the same seed source; individual plants from different seed sources; and pooled DNA from multiple plants from the same seed source. The three technical replicates (three samples from each *G. hirsutum* line TM-1, *G. barbadense* line 3-79, and their F_1_) showed negligible inconsistencies between runs with average similarity of 99.93% ± 0.0007. Therefore, allele calls are highly reproducible across runs. Individual plants from the same seed source showed a range of similarity from 100% for Coker 315 provided by CSIRO to 73.39% for Lu Mien 14 samples provided by CSIRO. A larger inconsistency was seen with duplicated samples from different seed sources. The amount of inconsistency varied considerably across different lines, from 66.61% for VIR-6615/MCU-5 samples provided by USDA-ARS (College Station) and CSIRO to 99.36% for Stoneville 474 samples provided by USDA-ARS (New Orleans) and CIRAD. When DNA of a pool of individual plants developed from the same seed source was analyzed, the percentage of similarity varied between lines from 79.48% for LuMien 14 to 99.84% for Sicot 189. Rates determined for individual lines and replications of different types are shown in Table 5.

**Table 5:** Percent similarities for technical and biological replicates of lines

Line	Percent Similarity				Percent Residual Heterozygosity in Pools
	Technical Replicates	Individual Plants		Line Pool	
		Same Seed Source	Diff. Seed Source		
TM-1	100.00*^a^*	—	89.77	—	—
3-79	99.90 (±0.0006)	—	—	—	—
F_1_(TM-1x3-79)	99.87 (±0.0006)	—	—	—	—
Coker 315	—	100.00	89.32	99.67 (±1.83E-5)	0.33
Delta Opal	—	97.86	—	96.16 (±0.0015)	3.51
Deltapine 16	—	96.23	95.54	94.62	4.96
Deltapine 90	—	99.74	95.68	99.47 (±0.0023)	0.57
LuMien 14	—	73.39	—	79.48 (±0.1688)	19.40
MCU-5	—	99.52 (±0.0020)	66.61	99.42 (±0.0019)	0.64
Namcala	—	97.17	—	95.92	2.72
Riverina Poplar	—	—	—	80.52	10.82
Sicala 40/Fibermax 966	—	—	97.59 (±0.0202)	99.25	0.57
Sicala V-2/Fibermax 989	—	—	—	99.60	0.38
Sicot 189	—	—	—	99.84	0.15
Sicot 71	—	—	—	98.92	1.06
Sicot 81	—	—	—	99.29	0.67
Sicot F-1	—	—	—	92.53	6.63
F_1_(TM-1x*im*)	—	99.97	—	—	—
F_1_(DP5690x*Li2*)	—	99.87	—	—	—
F_1_(STV474xHS26)	—	94.58	—	—	—
Acala Maxxa	—	—	88.16	—	—
Stoneville 474	—	—	99.36	—	—
Guazuncho II	—	—	89.19	—	—
Coker 312	—	—	84.20	—	—
Tamcot SP37	—	—	89.28	—	—

Standard deviations (SDs) are listed for comparisons with three samples; when no SD is listed, comparisons are between two samples.

a Complete identity, *i.e.*, no SD between the three samples.

### Genetic map construction

**Figure 5:**
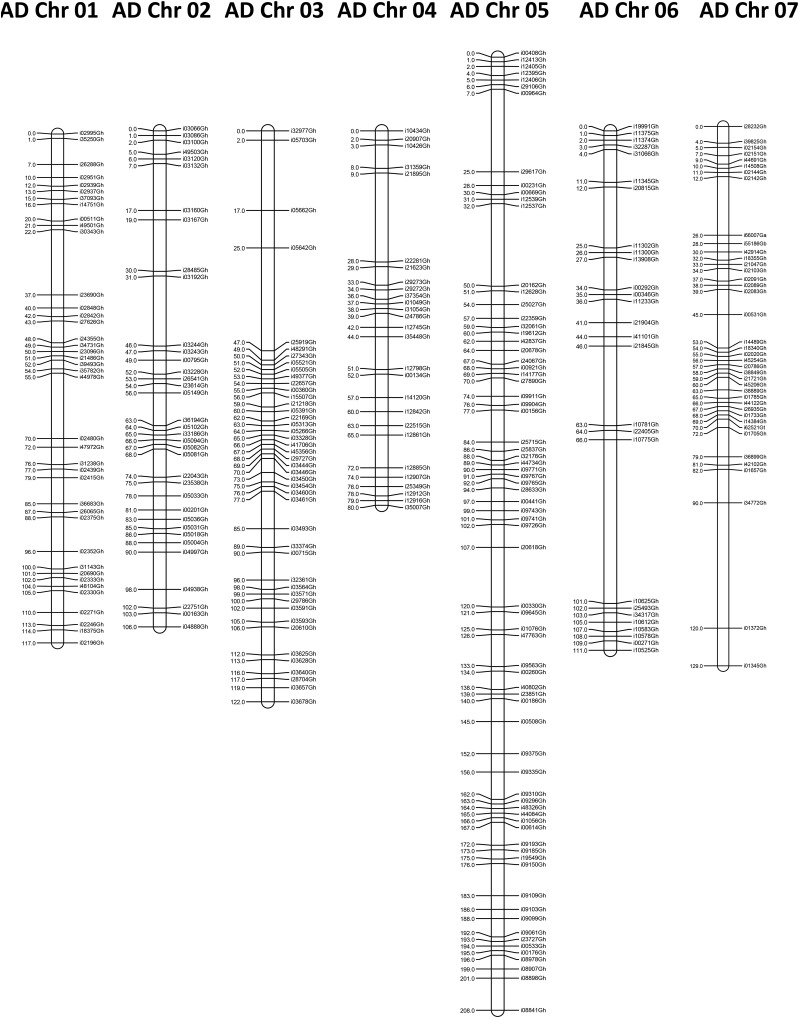

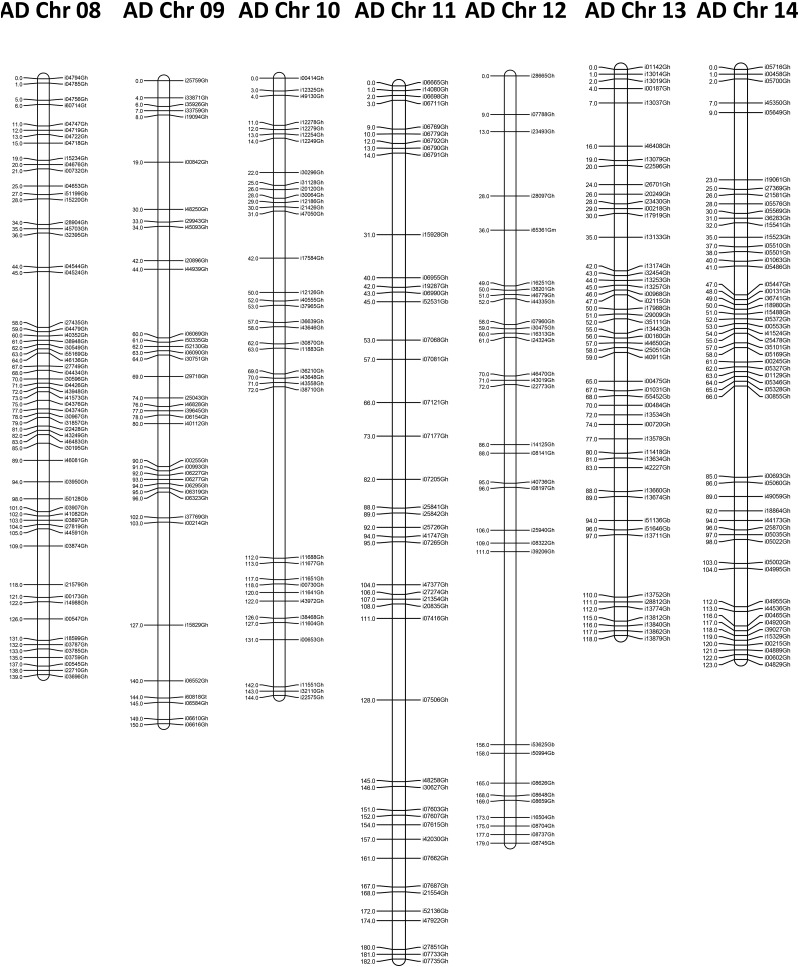

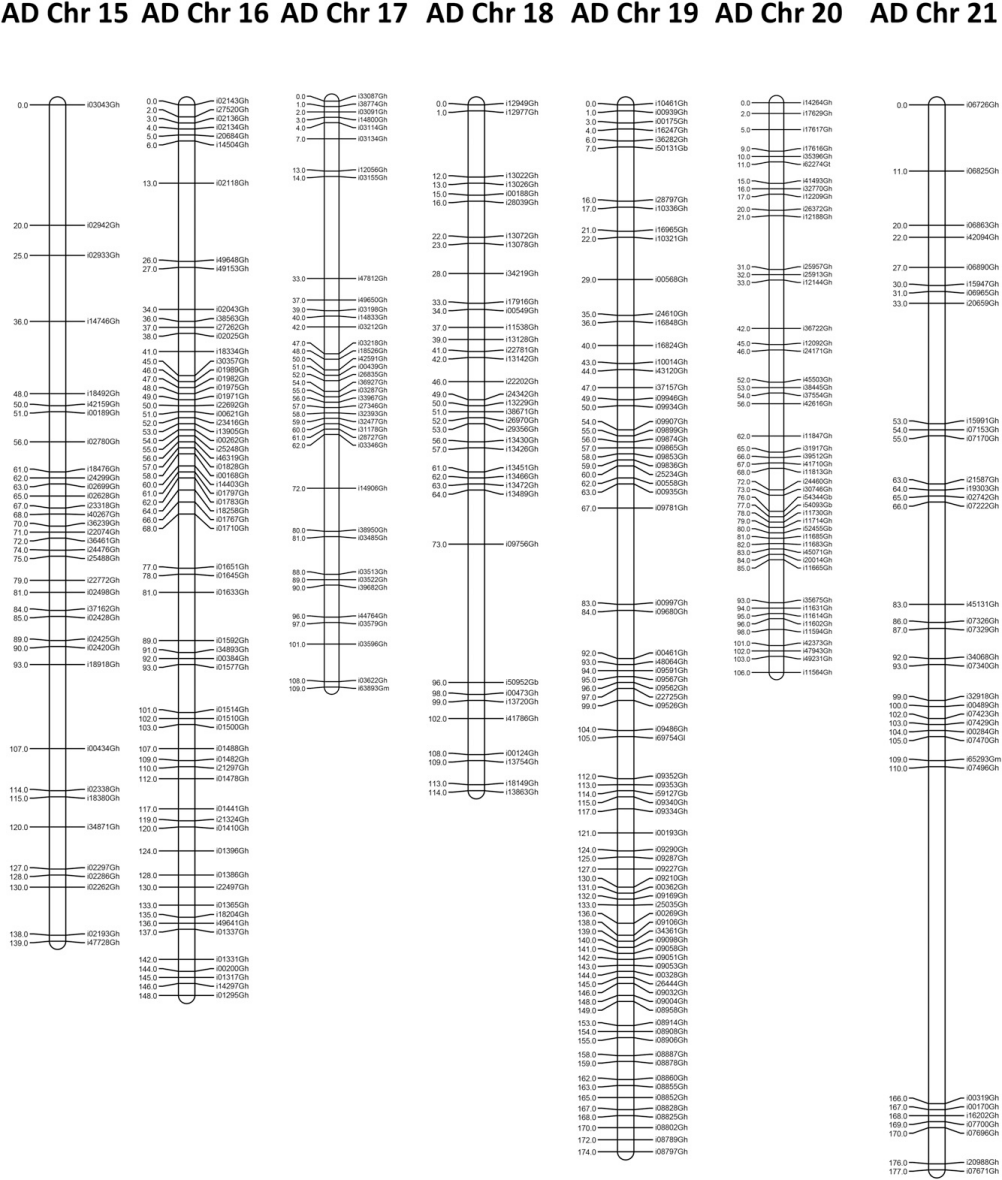

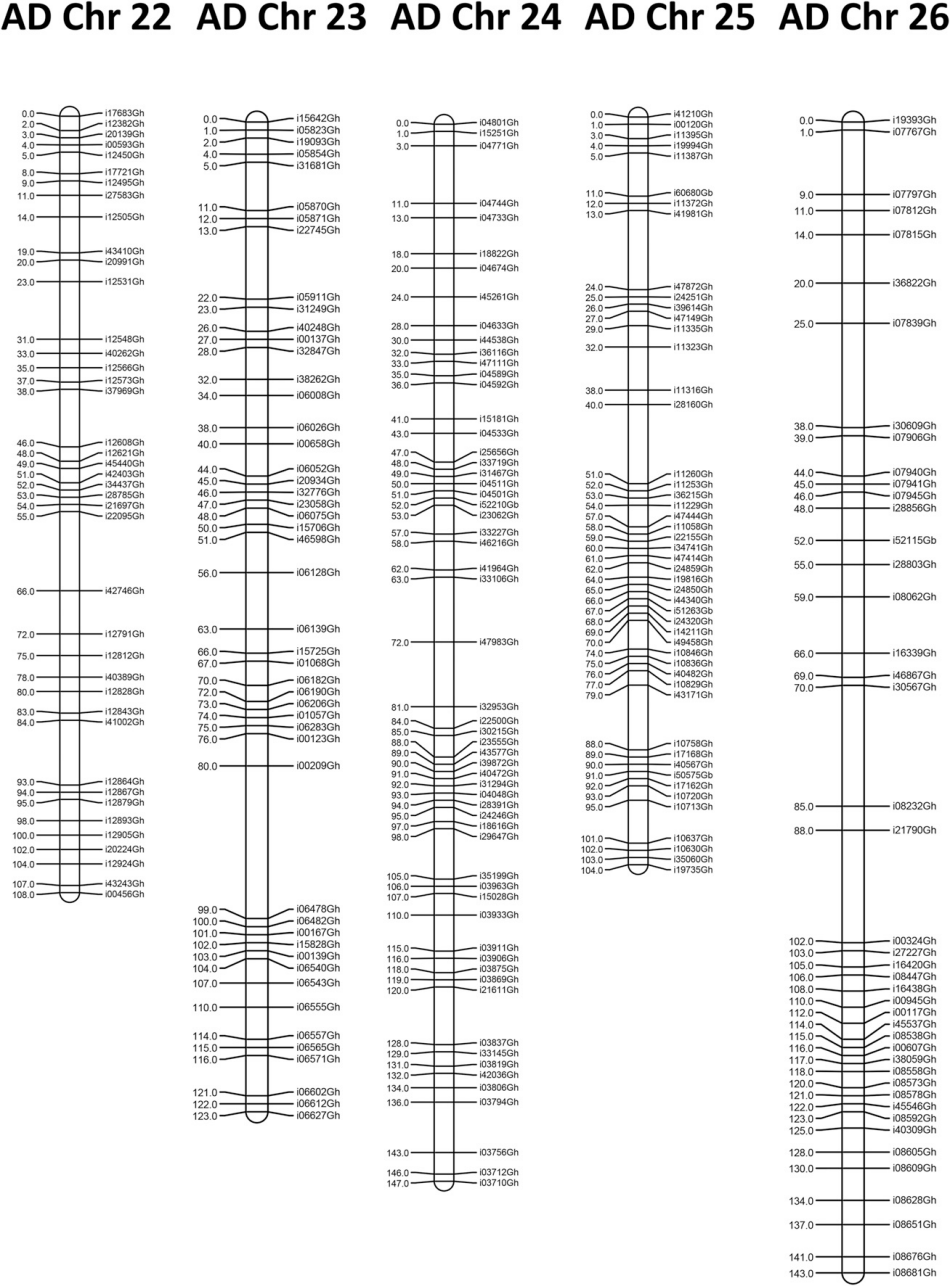
Intraspecific linkage map of 26 allotetraploid cotton chromosomes. Map determined using 93 F_2_ individuals from a Phytogen 72 by Stoneville 474 cross. Only one marker is listed on the right per Kosambi centiMorgan (cM) on the left, even if there were more markers co-segregating. Chromosomes are listed based on AD chromosome number.

An intraspecific map was generated from 93 F_2_ samples from a cross between *G. hirsutum* lines Phytogen 72 and Stoneville 474. A total of 7171 SNP markers were mapped in 26 linkage groups representing 3499 cM (Figure 5). These represent 6938 markers from the *G. hirsutum* set and 235 markers from the other species sets. An average of 254 markers per linkage group was mapped on A-subgenome chromosomes and 298 markers per linkage group of the D-subgenome. These correspond to an overall average of 55 bins with 4.98 markers/bin per chromosome, 51 bins with 4.59 markers/bin for A-subgenome chromosomes, and 60 bins with 5.40 markers/bin for D-subgenome chromosomes.

**Figure 6:**
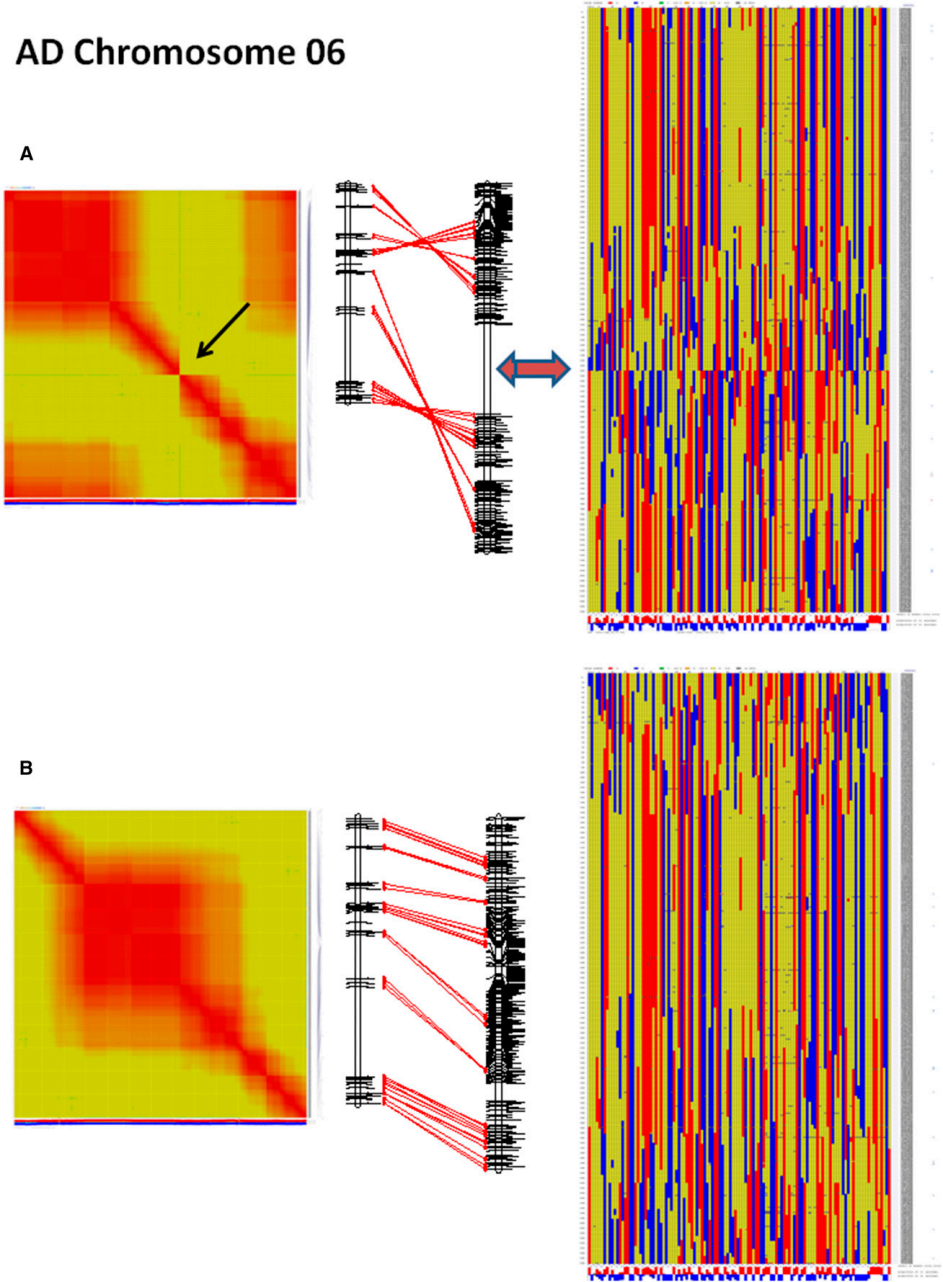
Inconsistencies between initial *de novo* interspecific map and the intraspecific map. (A) Initial plots of interspecific map order and correlation with intraspecific map show area of incorrect placement in center of the linkage group. (B) Corrected interspecific linkage group and final plots.

An initial interspecific map was generated using 118 F_2_ samples from a single interspecific cross between *G. barbadense* line 3-79 and *G. hirsutum* standard line TM-1. A total of 19,198 markers mapped into 26 linkage groups initially representing 4439.6 cM. Markers were largely distributed across the genome with a few moderately sized gaps. Correlation analysis between the initial interspecific map and the intraspecific map showed that the moderately sized gaps were associated with inverted marker orders compared to the intraspecific map in nine linkage groups corresponding to chromosomes: Chr06, Chr13, Chr15, Chr18, Chr20, Chr21, Chr22, Chr23, and Chr26. Linkage disequilibrium and recombination plots of these linkage groups showed incorrect ordering near the gaps (see Chr06 example in Figure 6). The most likely correct orientation is found in the intraspecific linkage groups, where the gaps are smaller. For example, in Figure 6, it is likely that the interspecific map is inverted due to low linkage across the gap but the intraspecific map does not have a corresponding gap and is likely the correct orientation. To correct this issue, the intraspecific map was used as a framework map to reorder the problematic interspecific linkage groups.

**Figure 7:**
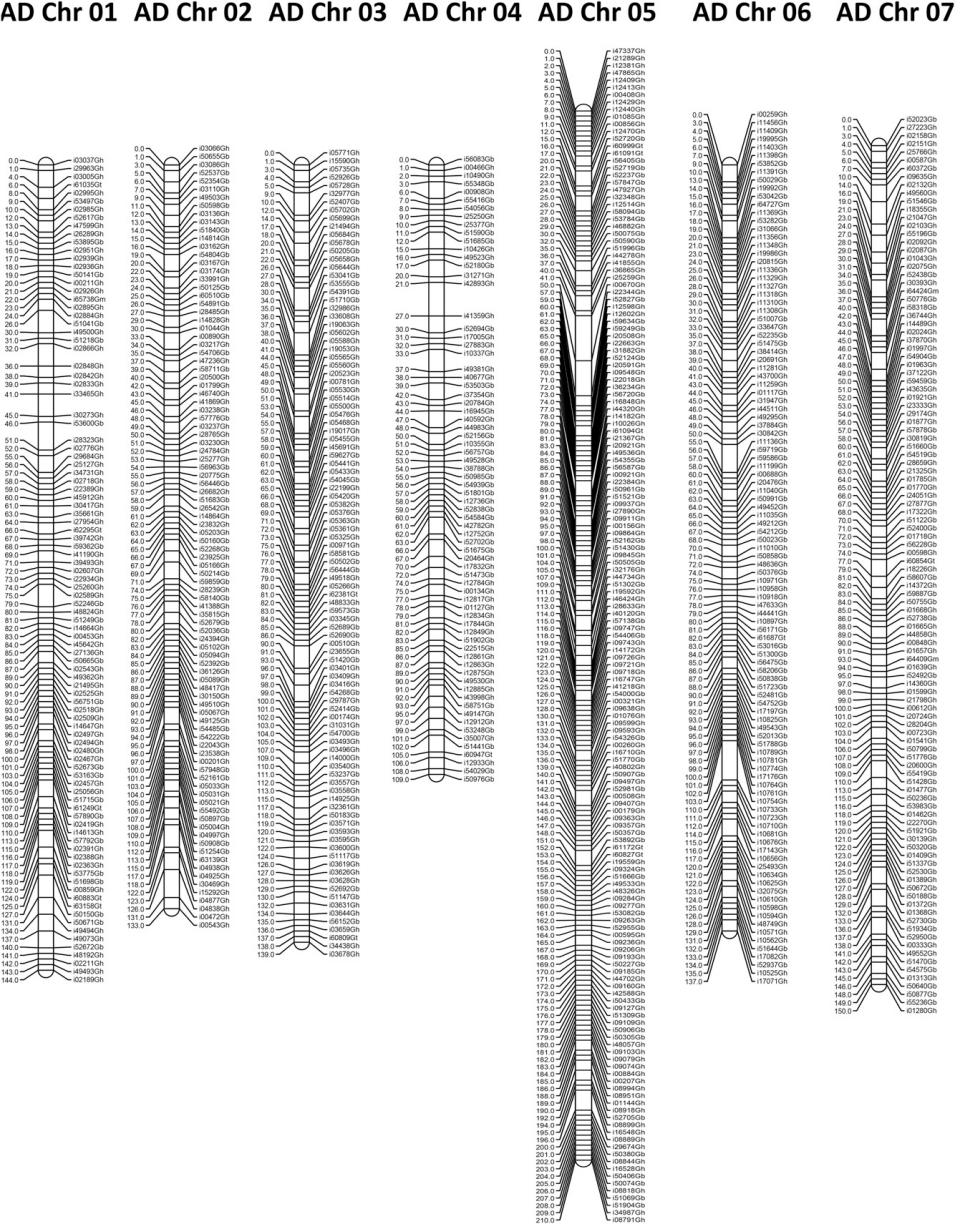

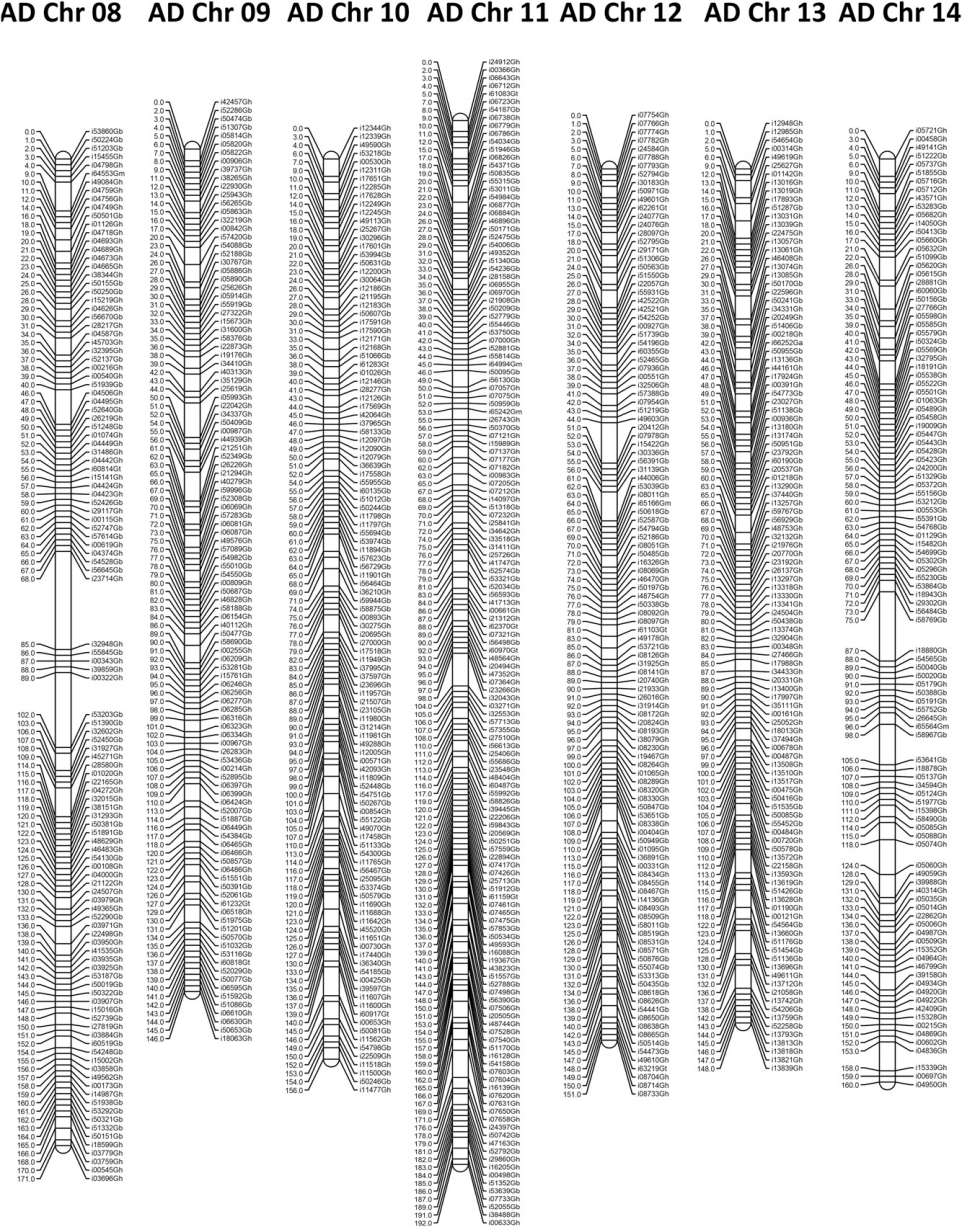

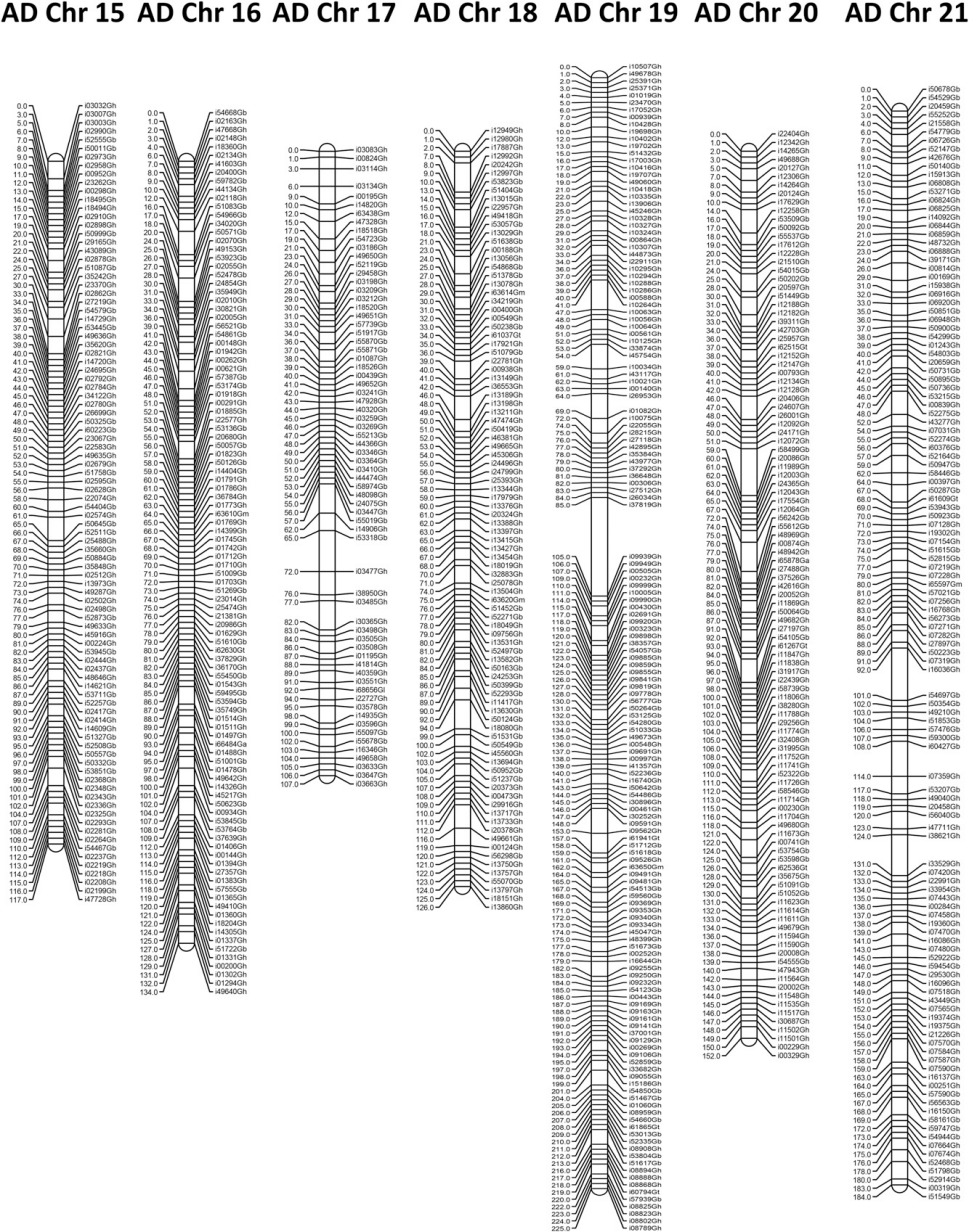

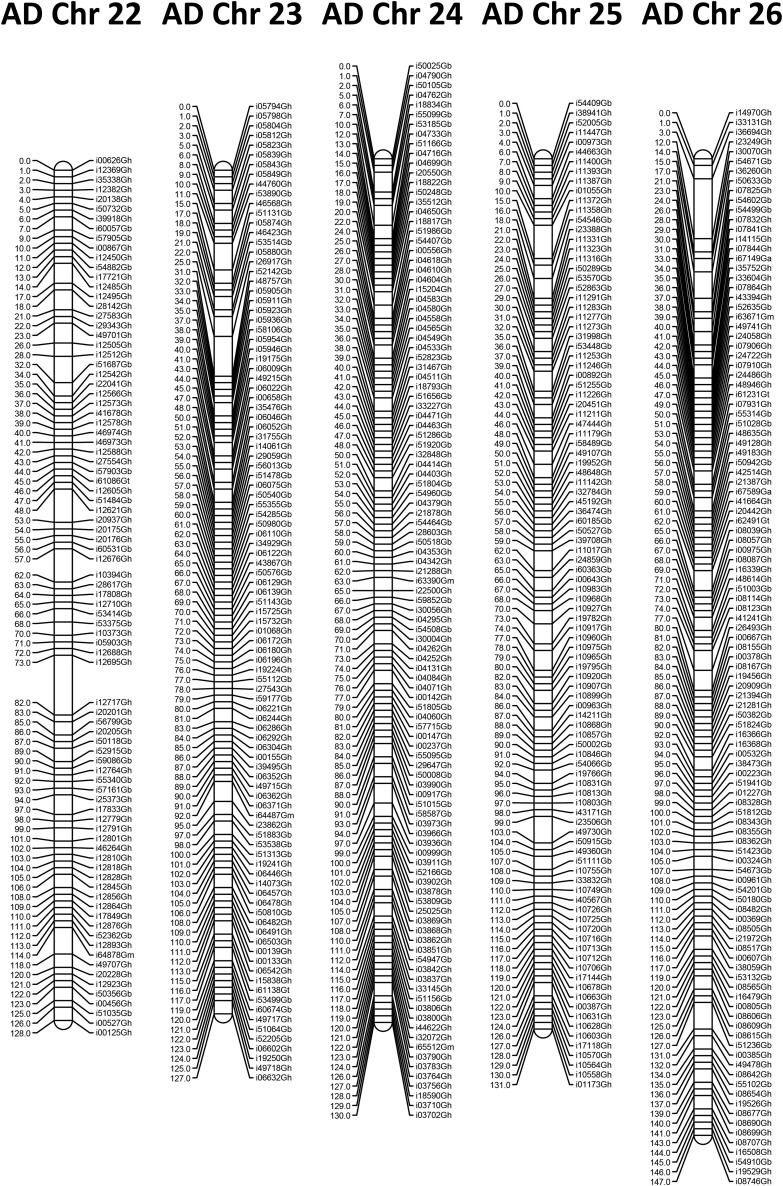
Interspecific linkage map of 26 allotetraploid cotton chromosomes. Map determined using 118 F_2_ individuals from a *G. barbadense* line 3-79 by *G. hirsutum* genetic standard line Texas Marker -1 cross. One marker listed on the right per Kosambi centiMorgan (cM) on the left. Chromosomes are listed based on AD chromosome number.

The final map includes the reordered linkage groups and contains 19,191 markers mapped to 26 linkage groups that collectively encompass 3854.3 cM (Figure 7). This map represents a reduction in size of 585.3 cM (13%) from the initial interspecific map. The mapped SNPs represent 11,452 markers from the *G. hirsutum* set and 7746 markers from the other species data sets (6785 for *G. barbadense*; 614 for *G. tomentosum*; 280 for *G. mustelinum*; 39 for *G. armourianum*; and 28 for *G. longicalyx*). The map contained an average of 162 recombination bins with 4.55 markers/bin per chromosome across the linkage groups, with an average of 166 bins with 4.52 markers per bin and 159 bins with 4.60 markers per bin in the A- and D-subgenome chromosomes, respectively. The average overall number of markers per linkage group was 738, with an average of 718 for A-subgenome chromosomes and 759 for D-subgenome chromosomes.

In the intraspecific and interspecific maps, the observed map lengths (cM) for the A-subgenome chromosomes (137.5 cM, 152.8 cM) were on average very similar to but slightly larger than those for the D-subgenome chromosomes (131.7 cM, 143.7 cM), respectively. For cotton chromosomes, accurate estimates and comparisons of genetic size with physical distance, *e.g.*, cM/Mbp, are not yet available. Accurate cytological estimates for individual allotetraploid chromosome sizes are not available. Although a BAC-by-BAC reference sequence for the D_5_ is available, highly repetitive regions are under-represented, including subtelomeric and large peri-centromeric regions. Therefore, a D_5_-based estimate would be limited in scope and would underestimate physical size. However, the genomes of the closely related extant diploids, *G. arboreum* and *G. raimondii*, have been estimated to be 1600 and 800 Mb, respectively; sizes of those genomes and chromosomes correspond well cytologically with absolute and relative sizes of chromosomes in the *G. hirsutum* subgenomes. Using the *G. arboreum* and *G. raimondii* estimates as a guide for A- and D-subgenome sizes, 1 cM in the intraspecific map represents an average of 895 kb in A-subgenome chromosomes and 467 kb in D-subgenome chromosomes. In the interspecific map, 1 cM represents an average physical distance of 805 kb in A-subgenome chromosomes and 428 kb in D-subgenome chromosomes. Although sizes and physical distances in A- and D-subgenome chromosomes differ approximately two-fold, the rate of crossing over does not, reflecting that most crossovers occur in nonheterochromatic regions or gene space.

Segregation distortion has been previously reported in cotton populations and, not unexpectedly, a number of markers in both the intraspecific and interspecific maps showed significant distortion from expected F_2_ segregation ratios. In the intraspecific map, 612 markers of the 7171 (8.5%) mapped markers showed significant segregation distortion (*P* < 0.05). Of these distorted markers, 256 (41.8%) show a decrease in amount of heterozygotes. Overall, when one parental allele is favored, generally an excess of the female parental allele (Phytogen 72) was observed at a 3.45-to-1 ratio. In the interspecific map, 3475 markers of the 19,191 (18.1%) mapped markers showed significant segregation distortion (*P* < 0.05). Unlike the intraspecific markers, when one parental allele is favored, there is not a large bias toward one parent, and a favored ratio of 1.13 is observed for a male parent plant (*G. hirsutum*) to female parent (*G. barbadense*) comparison. A skew toward the *G. barbadense* parent was observed in work by Lacape *et al.* (2003). The amounts of distorted markers in these populations are within the range of previous reported studies from 8.5% to more than 50% (Lacape *et al.* 2003; Shen *et al.* 2007).

### Distribution of recombination events

**Figure 8:**
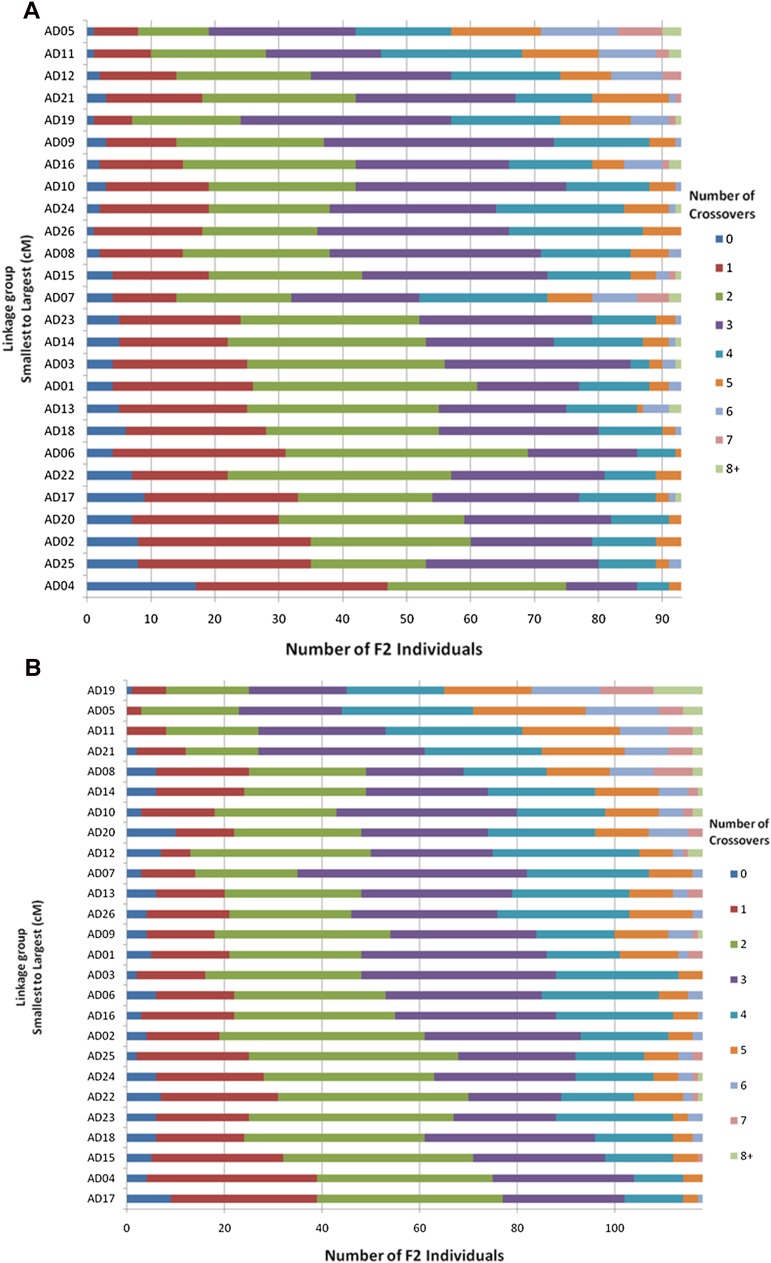
Frequency distribution of the number of crossovers. Numbers of crossovers detected for each F_2_ individual per chromosome (0 to >8) are displayed chromatically for each linkage group, which are organized by genetic size (longest at top, shortest at bottom). (A) Distribution of crossovers in the intraspecific mapping population. (B) Distribution of crossovers in the interspecific mapping population.

The high density of markers mapped in this study allowed the identification of almost all of the recombination events present in each F_2_ individual. We calculated the number of crossovers per F_2_ using all markers in both the intraspecific and interspecific linkage maps. In the intraspecific map, the number of crossovers across all chromosomes of an individual varied from 44 to 97, with an average of 67.5, ignoring two individuals with an abnormally high number of crossovers on a couple of linkage groups. These individuals were ignored; although they showed a normal number of crossovers across most chromosomes, they had much higher than two-times the number of crossovers as all other samples for one or more linkage groups. One individual had 34 crossovers on Chr13 and another individual had 24, 34, and 38 crossovers on Chr05, Chr19, and Chr14, respectively. An average of 2.65 crossovers per linkage group occurred across all individuals. Distribution of crossovers in the intraspecific population across linkage groups is shown in Figure 8A.

In the interspecific map, the number of crossovers per individual ranged from 54 to 97, with an average of 75.8 (ignoring a single individual that had an abnormally high number of crossovers in Chr14). Similar to the intraspecific mapping population, there was an average of 2.92 crossovers per linkage group across individuals. The distribution of crossovers in the interspecific population across linkage groups is shown in Figure 8B.

### Analyses of synteny

**Figure 9:**
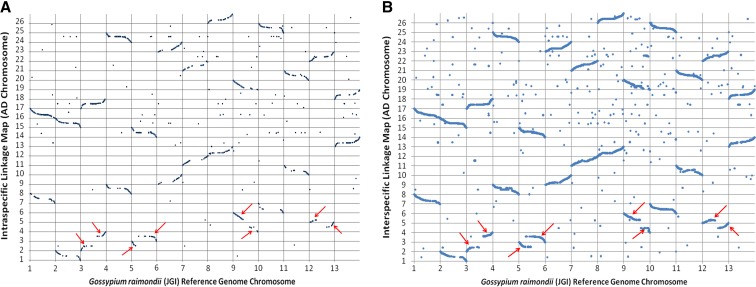
Dot plot of the syntenic positions of SNP markers in the allotetraploid linkage maps *vs.* the JGI *G. raimondii* reference genome. The 26 allotetraploid chromosomes are shown on the y-axis and the 13 chromosomes of *G. raimondii* are shown on the x-axis. Red arrows indicate translocation events relative to *G. raimondii*. (A) Intraspecific linkage map displaying positions of 4521 mapped SNP in *G. hirsutum* with alignments to *G. raimondii*. (B) Interspecific linkage map (*G. barbadense* line 3-79 by *G. hirsutum* genetic standard line Texas Marker -1) displaying positions of 12,027 mapped SNP with alignments to *G. raimondii*.

All of the 70,000 putative SNP markers that were used for production of the CottonSNP63K array were aligned to the D_5_ reference genome using BWA in Galaxy; 59.7% of the markers produced alignments to the reference. Synteny was detected for a total of 4521 and 12,027 mapped SNPs for the intraspecific and interspecific maps, respectively. Dot plots of the linkage maps vs. the D_5_ reference genome show high collinearity across linkage groups and chromosomes as expected, except for four A-subgenome chromosomes, which are known to contain translocations relative to D-chromosomes. Translocations involving chromosomes 2, 3, 4, and 5 were identified in both maps and are indicated in Figure 9, A and B.

When all markers were aligned to the A_2_ draft genome, 61.8% of the markers aligned to the reference. A total of 3863 and 11,344 SNPs that mapped were included in the intraspecific and interspecific maps, respectively. While a larger percentage of the SNPs mapped to A_2_ compared to D_5_, expected collinearity was not observed when these A_2_ alignment positions were plotted against position in the linkage maps (Figure S3, A and B), as was seen with the high-quality BAC-by-BAC–derived D_5_ reference genome. The strong collinearity with the high-quality D_5_ genome suggests that our genetic maps are correctly ordered, whereas the low collinearity with the A_2_ genome sequence suggests that the genome sequence may be improved using the maps produced in this effort and other genetic maps.

## Discussion

The interspecific and intraspecific genetic maps developed with the CottonSNP63K represent the most saturated maps developed for cotton to date. The produced intraspecific genetic map is the first saturated map for a cross between two *G. hirsutum* lines. While one other effort has come close to generating a number of linkage groups equal to the 26 cotton chromosomes (Zhang *et al.* 2009), to the best of our knowledge the intraspecific map developed here is the first that associates into 26 linkage groups corresponding to the number of cotton chromosomes. The array and maps provide a foundation for fine mapping and genetic dissection of agronomically and economically important traits, and facilitate the identification of causative genes underlying quantitative trait loci. This new tool will also foster map-based cloning and genome assembly efforts and contribute to advancements in marker-assisted selection and genomic selection in breeding programs. Although our initial interspecific map was very close to the estimated tetraploid map size of ∼4500 cM suggested by Rong *et al.* (2004), it showed sizeable gaps and discordant LD patterns. Once corrected, the size of both maps was well below the estimated value of ∼4500 cM at 3854.3 cM for the interspecific map and 3499 cM for the intraspecific map. A decrease in map size is expected when higher-density maps are generated, due to the improved ability to distinguish scoring errors from double recombinant events with high-density markers (Rong *et al.* 2004). Although the sizes of the new interspecific and intraspecific maps are lower than expected, they both fall well within the range of previous map sizes of published interspecific maps 3380 to 5115 cM (Yu *et al.* 2012; Blenda *et al.* 2012; Shi *et al.* 2014) and within the range of 2061 to 4448 cM for reported intraspecific maps (Zhang *et al.* 2012; Gore *et al.* 2014; Rong *et al.* 2004; Tang *et al.* 2014).

The mapping of a large number of markers from different discovery sets utilizing a large variety of different cotton lines, primarily cultivars as shown in Table 2, along with a moderate percentage of markers showing intermediate to high MAFs suggests high transferability of markers across different sets of cotton germplasm. To have a wide applicability across cotton samples, the array was designed to provide the most comprehensive tool to date for cotton researchers and included SNPs that were discovered from technologies targeting both gene regions and genomic regions. This approach was applied so that the inclusion of markers outside of genes would generate a more even distribution across chromosomes because genic markers are unevenly distributed across chromosomes (Hulse-Kemp *et al.* 2014). Because the majority of the gene-based markers were provided by the CSIRO discovery set using the D_5_ sequence assembly as a reference (Zhu *et al.* 2014), many A-subgenome-specific genes may be under-represented in the gene-based marker set. Fortunately, genomic markers located in LD with a gene of interest can also be of value in genome-wide association studies, and thus help compensate for the subgenomic bias among genic intraspecific markers. As the TAMU/UC-Davis Intra-Genomic Set 1 and TAMU/UC-Davis Inter-Genomic sets are markers derived from BAC-end sequences (Hulse-Kemp *et al.* 2015), these markers will provide an avenue for direct map-based cloning and fine-mapping of identified regions of interest through direct integration of the array with BAC-based resources.

The array provides cotton research groups with a standardized set of markers that can be used to localize and replicate important previously identified markers, and it will also allow for investigating different types of sample inconsistencies. The replication analysis performed here showed that while technical DNA replicates show very high consistency, different levels of variability were seen with other replication types. As found in maize (Yan *et al.* 2009), the array analysis revealed considerable variation within some cotton lines, not only between different seed sources but also within the same seed source. Notably, two different TM-1 samples, typically used as a *G. hirsutum* genetic standard line (Kohel, Richmond, and Lewis 1970), show only 89.77% similarity. Hinze *et al.* (2015) found similar inconsistencies using SSRs in the US National Cotton Germplasm Collection where accessions with a common name may or may not have been identical whereas other accessions with no reason for similarity were genetically identical. Such inconsistencies can create difficulties when trying to interpret or compare studies utilizing samples bearing a common name. The CottonSNP63K provides an efficient way to characterize these inconsistencies because it has negligible inconsistencies with reproducibility; thus, the similarity differences discovered between seed sources are due to variation of the sources, not a problem with genotyping on the array. The amount of residual heterozygosity within the same seed source shows that some lines are more heterogeneous than others, and comparisons across studies using heterogeneous lines will be less reproducible. However, residual heterozygosity in lines also will offer direct avenues for fine mapping of genes in primarily isogenic backgrounds (Truco *et al.* 2013), as well as provide additional diversity within *G. hirsutum* that has been noted to have low levels of diversity (Tyagi *et al.* 2014).

Due to the low diversity among cotton lines, multiple mapping populations will be required to map all of the polymorphic SNPs on the CottonSNP63K array. Analysis of the two mapping populations reported here allowed us to map a total of 22,829 total markers, with 3533 SNPs being placed into both linkage maps. Classification of SNPs into the intraspecific or interspecific groups was based on which of our data sets the SNP was identified in. The SNP classification does not indicate if it will or will not be a useful marker in other germplasm types. For example, 3533 of the SNP loci are dually represented in the intraspecific and the interspecific maps reported here. Utilizing mapped loci will be very straightforward, but utilizing the other markers will be more difficult, at least until they are mapped. Syntenic analyses with available high-quality related genome sequences such as the JGI D_5_ genome provide some insight into localization information in tetraploid cotton (Paterson *et al.* 2012). Allotetraploid linkage groups determined here showed a linear alignment with the D_5_ genome (Figure 9, A and B) and were also able to identify historic reciprocal translocation events between allotetraploid chromosomes 2/3 and 4/5 (Desai *et al.* 2006). The breakpoints for the translocations can be roughly mapped to homeologous regions with the linkage maps to between 21.7 and 29.5 Mb in D_5_ chromosome 3 and between 16.7 and 21 Mb in D_5_ chromosome 5 for allotetraploid chromosomes 2/3, between 40.1 and 46.1 Mb in D_5_ chromosome 9, and between 11.5 and 23.2 Mb in D_5_ chromosome 12 for allotetraploid chromosomes 4/5. It has been suggested that these translocations involved complete arms (Blenda *et al.* 2012), which is not ostensibly discordant to the approximate breakpoints found here. However, additional mapping would be required to pinpoint exact locations relative to the D_5_ chromosomes because map information near the breakpoints is still ambiguous and overlapping at points.

Like SNP arrays developed for other crops, we found a relatively large number of markers that were difficult to score and required either manual adjustment of markers or eliminating the marker from the analysis completely. Compared to diploids, the success rates for SNPs in arrays are typically lower for polyploids due to the presence of duplicated loci in homeologous, paralagous regions, the low levels of divergence particularly between gene copies in different subgenomes, and also between paralagous regions in the same subgenomes. The 61.6% success rate found for the CottonSNP63K is quite comparable to the success rates of arrays generated for other polyploid crops, such as 61% for oat (Tinker *et al.* 2014) and 63% for wheat (Wang *et al.* 2014). Most of the markers mapped in this study were localized to a single map position, as was also the case for the majority of markers mapped with the wheat 90K array (Wang *et al.* 2014), indicating that most SNP assays are specific to a single locus and/or assaying segregation of a single locus. Similarly, we also found that success rates among discovery sets included on the array were highly variable (Table 4). The differences in success rates for discovery efforts as determined by this effort can provide insights for future SNP development efforts by examining parameters and pipelines used and the outcome of those discovery sets. The information provided by the array will enable future SNP discovery efforts in cotton to focus on increasing the success rate of SNP predictions.

## Conclusions

The CottonSNP63K and the accompanying cluster file provide a standardized high-throughput genotyping tool for the cotton community with limited ascertainment bias over a wide range of cotton germplasm. The high-density genetic maps and their synteny with the *G. raimondii* reference genome provide a useful resource for analyzing genome-wide variation in allotetraploid cotton. Once the allotetraploid cotton genome sequence is available, these maps will be a resource to validate and refine the genome assemblies, as well as provide a resource to directly integrate physical and genetic resources. The array and maps provide a foundation for the genetic dissection of agronomically and economically important traits and crop improvement through genomics-assisted selection. It will also foster positional cloning and genome assembly efforts. Efforts are underway to also provide a comprehensive diversity analysis of a wide range of cotton material, because the results will further extend utility of the array, component SNPs, and associated genomics resources.

## Supplementary Material

Supporting Information
