# Supplementary material for: Development of a 63K SNP Array for Cotton and High-Density Mapping of Intraspecific and Interspecific Populations of Gossypium spp
Source: G3 (Bethesda). 2015 Apr 22;5(6):1187–209. doi: 10.1534/g3.115.018416 (PMC4478548; doi:10.1534/g3.115.018416)
Supplement: Supporting Information [file supp_5_6_1187__index.html]

Development of a 63K SNP Array for Cotton and High-Density Mapping of Intraspecific and Interspecific Populations of Gossypium spp. — Supporting Information 

# Development of a 63K SNP Array for Cotton and High-Density Mapping of Intraspecific and Interspecific Populations of *Gossypium* spp.

## Supporting Information for Hulse-Kemp *et al.*, 2015

**Files in this Data Supplement:**

- Supporting Information - Figure S1 and Tables S1-S2 (PDF, 450 KB)
- Figure S1 - Dot plot of the syntenic positions of SNP markers in the allotetraploid linkage maps versus the BGI *G. arboreum* draft genome. (PDF, 1 MB)
- Table S1 - Excel spreadsheet listing all 70K markers and their descriptions on the CottonSNP63K. (.xlsx, 13 MB)
- Table S2 - Excel spreadsheet showing results of assay design of discovery group sets using Illumina's Assay Design Tool. (.xlsx, 11 KB)
