# Supplementary material for: Development of a 63K SNP Array for Cotton and High-Density Mapping of Intraspecific and Interspecific Populations of Gossypium spp
Source: G3 (Bethesda). 2015 Apr 22;5(6):1187–209. doi: 10.1534/g3.115.018416 (PMC4478548; doi:10.1534/g3.115.018416)
Supplement: Supporting Information [file supp_g3.115.018416_018416SI.pdf]

G3: Genes | Genomes | Genetics

Development of a 63K SNP Array for Cotton and High-density Mapping of Intra- and Inter-specific Populations of *Gossypium* spp.

Amanda M. Hulse-Kemp[1,2], Jana Lemm[3], Joerg Plieske[3], Hamid Ashrafi[4], Ramesh Buyyarapu[5], David D. Fang[6], James Frelichowski[7], Marc Giband[8,9], Steve Hague[1], Lori L. Hinze[7], Kelli J. Kochan[10], Penny K. Riggs[10,2], Jodi A. Scheffler[11], Joshua A. Udall[12], Mauricio Ulloa[13], Shirley S. Wang[7], Qian-Hao Zhu[14], Sumit K. Bag[15], Archana Bhardwaj[15], John J. Burke[13], Robert L. Byers[12], Michel Claverie[8], Michael A. Gore[16], David B. Harker[12], Md S. Islam[6], Johnie N. Jenkins[17], Don C. Jones[18], Jean-Marc Lacape[8], Danny J. Llewellyn[14], Richard G. Percy[7], Alan E. Pepper[19,2], Jesse A. Poland[20], Krishan Mohan Rai[15], Samir V. Sawant[15], Sunil Kumar Singh[15], Andrew Spriggs[14], Jen M. Taylor[14], Fei Wang[1], Scott M. Yourstone[12], Xiuting Zheng[1], Cindy T. Lawley[21], Martin W. Ganal[3], Allen Van Deynze[4], Iain W. Wilson[14], David M. Stelly[1,2,22]

[1]Department of Soil & Crop Sciences, Texas A&M University, College Station, Texas 77843, USA
[2]Interdisciplinary Degree Program in Genetics, Texas A&M University, College Station, Texas 77843, USA
[3]TraitGenetics GmbH, 06466 Gatersleben, Germany
[4]Department of Plant Sciences and Seed Biotechnology Center, University of California-Davis, Davis, California 95616, USA
[5]Dow AgroSciences, Trait Genetics & Technologies, Indianapolis, Indiana 46268, USA
[6]USDA-ARS-SRRC, Cotton Fiber Bioscience Research Unit, New Orleans, Louisiana 70124, USA
[7]USDA-ARS-SPARC, Crop Germplasm Research Unit, College Station, Texas 77845, USA
[8]CIRAD, UMR AGAP, Montpellier, F34398, France
[9]EMBRAPA, Algodão, Nucleo Cerrado, 75.375-000 Santo Antônio de Goias, GO, Brazil
[10]Department of Animal Science, Texas A&M University, College Station, Texas, 77843, USA
[11]USDA-ARS, Jamie Whitten Delta States Research Center, Stoneville, Mississippi 38776, USA
[12]Brigham Young University, Plant and Wildlife Science Department, Provo, Utah 84602, USA
[13]USDA-ARS, PA, Plant Stress and Germplasm Development Research Unit, Lubbock, Texas 79415, USA
[14]CSIRO Agriculture Flagship, Black Mountain Laboratories, ACT 2601, Australia
[15]CSIR-National Botanical Research Institute, Plant Molecular Biology Division, Lucknow-226001, UP, India
[16] Plant Breeding and Genetics Section, School of Integrative Plant Science, Cornell University, Ithaca, New York, 14853, USA
[17]USDA-ARS, Genetics and Precision Agriculture Research, Missississippi State, Mississippi, 39762, USA
[18]Cotton Incorporated, Agricultural Research, Cary, North Carolina, 27513, USA
[19]Department of Biology, Texas A&M University, College Station, Texas, 77843, USA
[20]Wheat Genetics Resource Center, Department of Plant Pathology and Department of Agronomy, Kansas State University, Manhattan, Kansas, 66506, USA
[21]Illumina Inc., 499 Illinois Street, San Francisco, California, 94158, USA
[22]Corresponding Author, email: stelly@tamu.edu

Cotton sequences utilized for array construction are available at: http://www.cottongen.org

Corresponding author: David M. Stelly, Texas A&M University, 370 Olsen Blvd., 2474 TAMU, College Station, TX 77843-2474. 979-845-2745 (Phone). 979-845-0456 (Fax). stelly@tamu.edu.

DOI: 10.1534/g3.115.018416

A

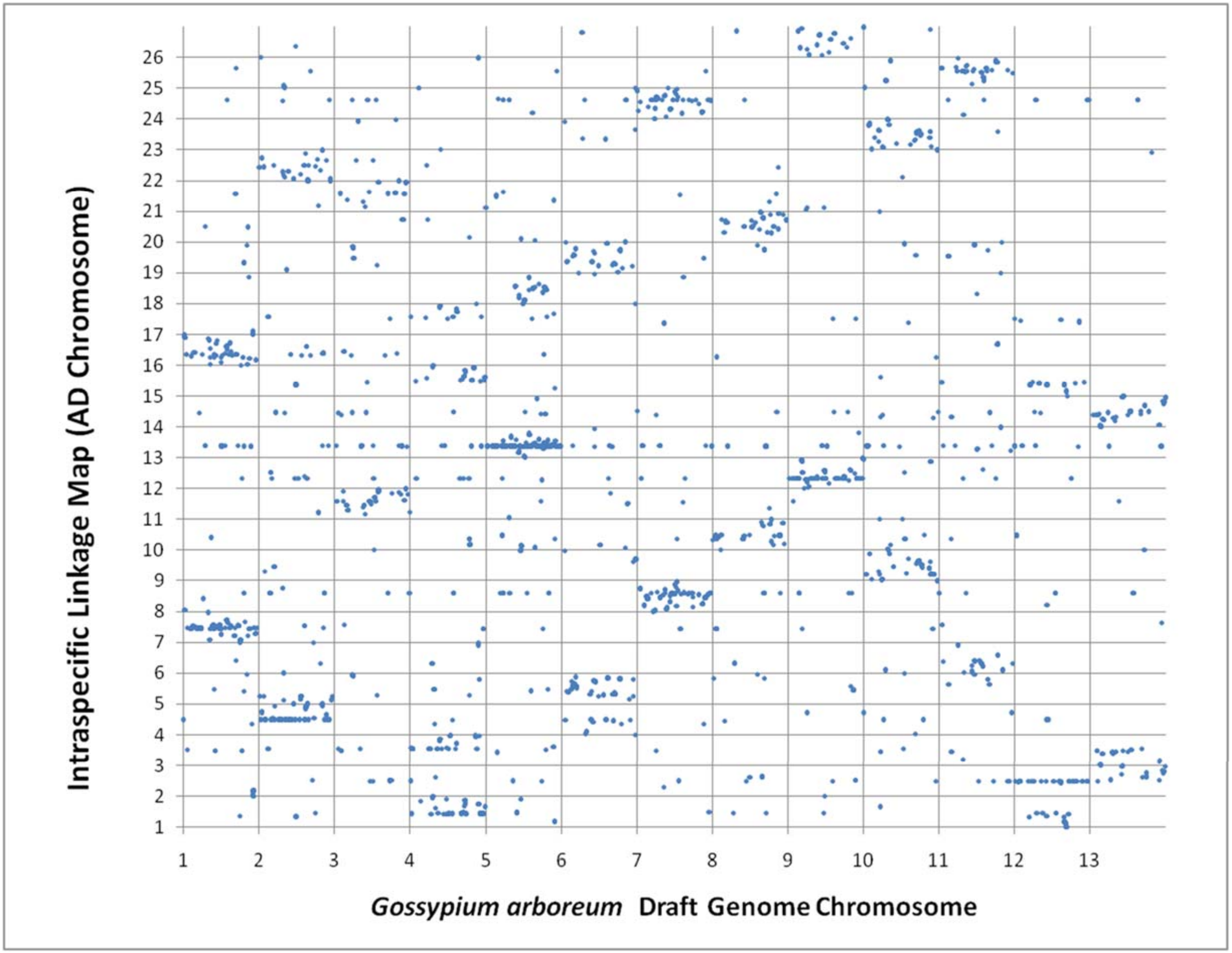

Intraspecific Linkage Map (AD Chromosome)
Gossypium arboreum Draft Genome Chromosome

B

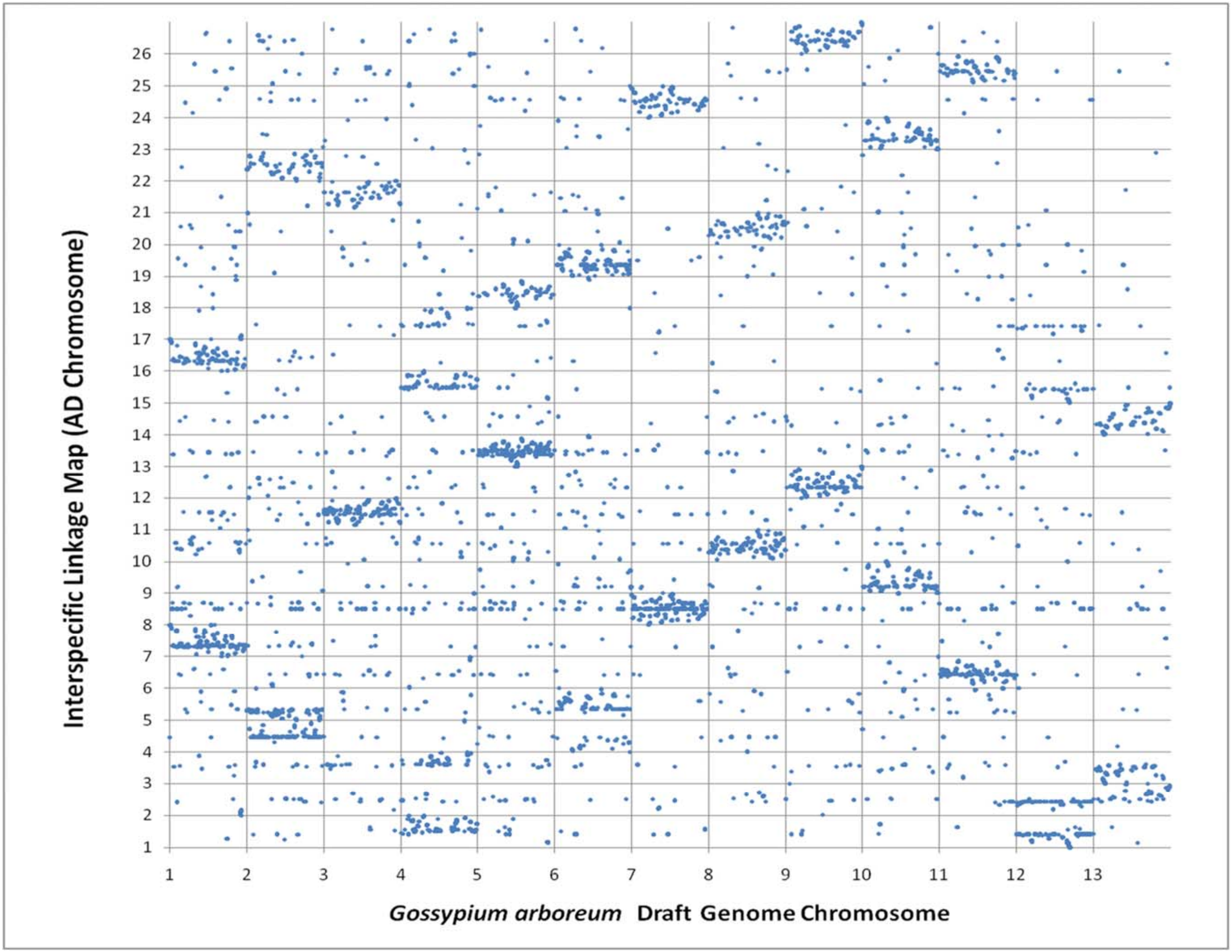

Interspecific Linkage Map (AD Chromosome)
26
25
24
23
22
21
20
19
18
17
16
15
14
13
12
11
10
9
8
7
6
5
4
3
2
1
1 2 3 4 5 6 7 8 9 10 11 12 13
Gossypium arboreum Draft Genome Chromosome

Figure S1 Dot plot of the syntenic positions of SNP markers in the allotetraploid linkage maps versus the BGI *G. arboreum* draft genome. The 26 allotetraploid chromosomes are shown on the y-axis and the 13 chromosomes of *G. arboreum* are shown on the x-axis. A.) Intra-specific linkage map displaying positions of 3,863 mapped SNP in *G. hirsutum* with alignments to *G. arboreum*. B.) Inter-specific linkage map (*G. hirsutum* genetic standard line Texas Marker -1 by *G. barbadense* line 3-79) displaying positions of 11,344 mapped SNP with alignments to *G. arboreum*.

Tables S1-S2

Available for download at www.g3journal.org/lookup/suppl/doi:10.1534/g3.115.018416/-/DC1

Table S1 Excel spreadsheet listing all 70K markers and their descriptions on the CottonSNP63K.

Table S2 Excel spreadsheet showing results of assay design of discovery group sets using Illumina's Assay Design Tool.
