# Supplementary material for: Development of a 63K SNP Array for Cotton and High-Density Mapping of Intraspecific and Interspecific Populations of Gossypium spp
Source: G3 (Bethesda). 2015 Apr 22;5(6):1187–209. doi: 10.1534/g3.115.018416 (PMC4478548; doi:10.1534/g3.115.018416)
Supplement: Supporting Information [file supp_g3.115.018416_FigureS1.pdf]

A

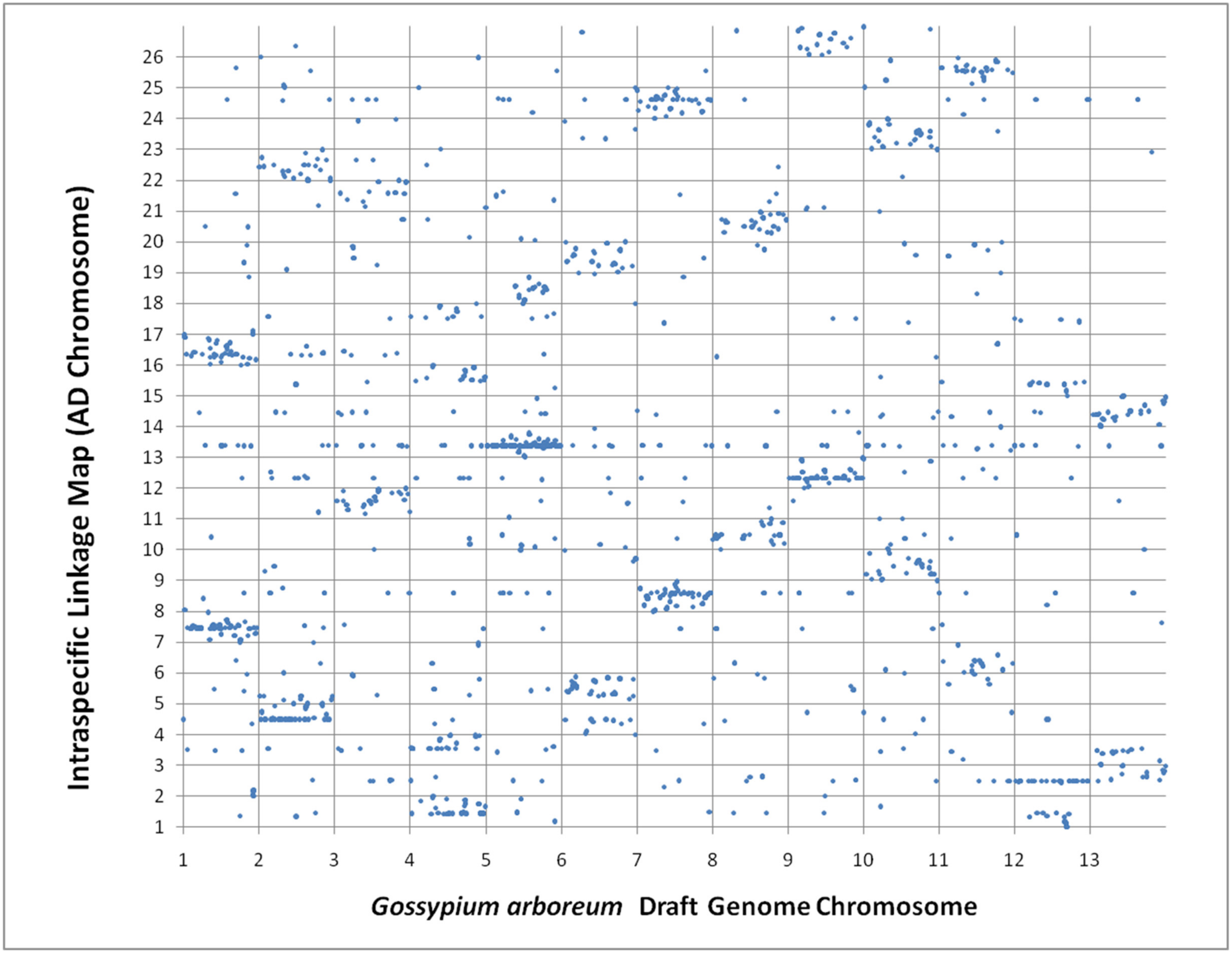

Intraspecific Linkage Map (AD Chromosome)
26
25
24
23
22
21
20
19
18
17
16
15
14
13
12
11
10
9
8
7
6
5
4
3
2
1
1
2
3
4
5
6
7
8
9
10
11
12
13
Gossypium arboreum Draft Genome Chromosome

B

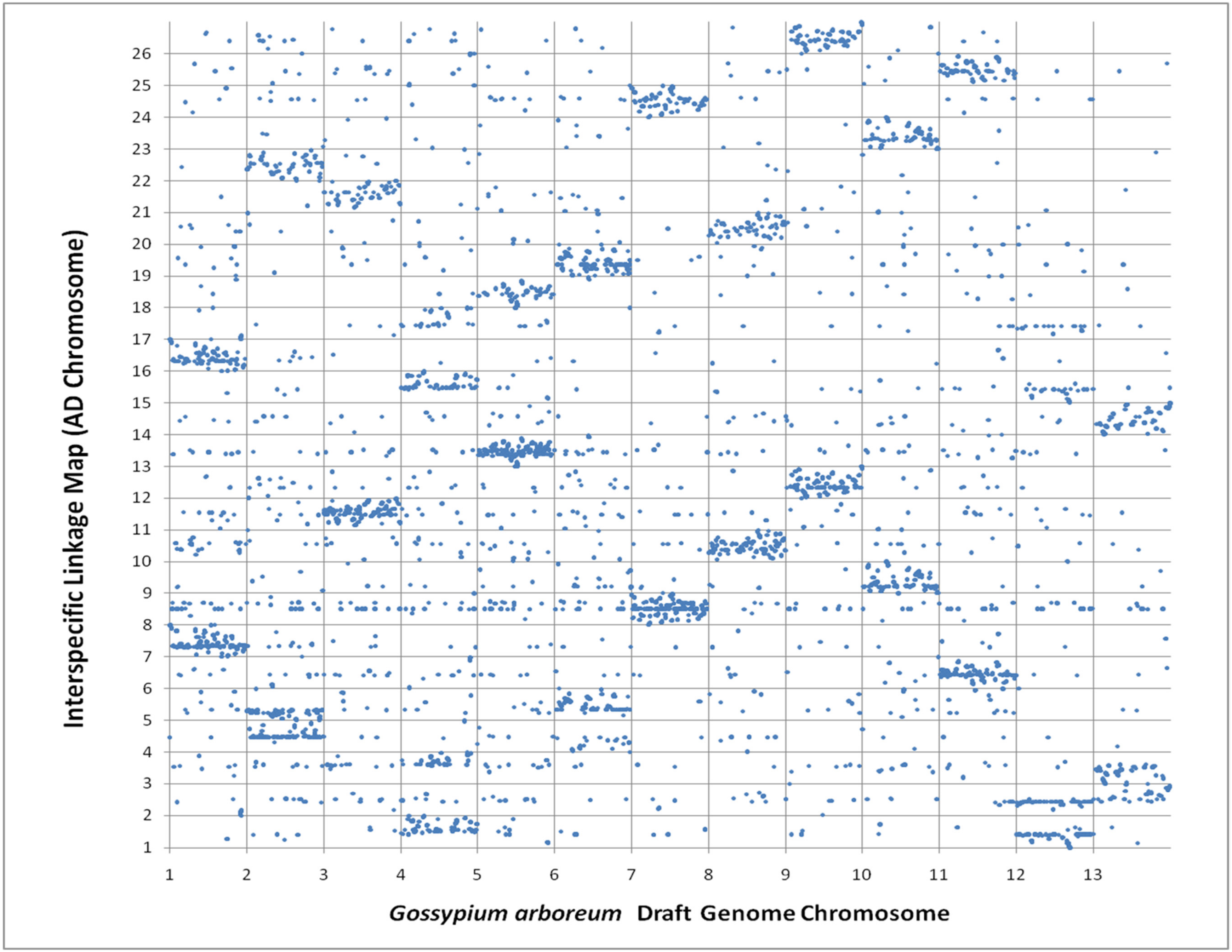
Interspecific Linkage Map (AD Chromosome)
26
25
24
23
22
21
20
19
18
17
16
15
14
13
12
11
10
9
8
7
6
5
4
3
2
1
1
2
3
4
5
6
7
8
9
10
11
12
13
Gossypium arboreum Draft Genome Chromosome

Figure S1 Dot plot of the syntenic positions of SNP markers in the allotetraploid linkage maps versus the BGI *G. arboreum* draft genome. The 26 allotetraploid chromosomes are shown on the y-axis and the 13 chromosomes of *G. arboreum* are shown on the x-axis. A.) Intra-specific linkage map displaying positions of 3,863 mapped SNP in *G. hirsutum* with alignments to *G. arboreum*. B.) Inter-specific linkage map (*G. hirsutum* genetic standard line Texas Marker -1 by *G. barbadense* line 3-79) displaying positions of 11,344 mapped SNP with alignments to *G. arboreum*.
